# Prevalence, intensity and associated risk factors of soil-transmitted helminth and schistosome infections in Kenya: Impact assessment after five rounds of mass drug administration in Kenya

**DOI:** 10.1371/journal.pntd.0008604

**Published:** 2020-10-07

**Authors:** Collins Okoyo, Suzy J. Campbell, Katherine Williams, Elses Simiyu, Chrispin Owaga, Charles Mwandawiro

**Affiliations:** 1 Eastern and Southern Africa Centre of International Parasite Control, Kenya Medical Research Institute (KEMRI), Nairobi, Kenya; 2 School of Mathematics, College of Biological and Physical Sciences, University of Nairobi, Nairobi, Kenya; 3 Deworm the World, Evidence Action, Washington DC, United States of America; 4 Deworm the World, Evidence Action, Nairobi, Kenya; World Health Organization, SWITZERLAND

## Abstract

**Background:**

In Kenya, over five million school age children (SAC) are estimated to be at risk of parasitic worms causing soil-transmitted helminthiasis (STH) and schistosomiasis. As such, the Government of Kenya launched a National School Based Deworming (NSBD) program in 2012 targeting the at-risk SAC living in endemic regions, with the aim of reducing infections prevalence to a level where they no longer constitute a public health problem. The impact of the program has been consistently monitored from 2012 to 2017 through a robust and extensive monitoring and evaluation (M&E) program. The aim of the current study was to evaluate the parasitological outcomes and additionally investigate water, sanitation and hygiene (WASH) related factors associated with infection prevalence after five rounds of mass drug administration (MDA), to inform the program’s next steps.

**Materials and methods:**

We utilized a cross-sectional design in a representative, stratified, two-stage sample of school children across six regions in Kenya. A sample size of 100 schools with approximately 108 children per school was purposively selected based on the Year 5 STH infection endemicity prior to the survey. Stool samples were examined for the presence of STH and *Schistosoma mansoni* eggs using double-slide Kato-Katz technique, urine samples were processed using urine filtration technique for the presence of *S*. *haematobium* eggs. Survey questionnaires were administered to all the participating children to collect information on their demographic and individual, household and school level WASH characteristics.

**Principal findings:**

Overall, STH prevalence was 12.9% (95%CI: 10.4–16.1) with species prevalence of 9.7% (95%CI: 7.5–12.6) for *Ascaris lumbricoides*, 3.6% (95%CI: 2.2–5.8) for *Trichuris trichiura* and 1.0% (95%CI: 0.6–1.5) for hookworm. *S*. *mansoni* prevalence was 2.2% (95%CI: 1.2–4.3) and *S*. *haematobium* prevalence was 0.3% (95%CI: 0.1–1.0). All the infections showed significant prevalence reductions when compared with the baseline prevalence, except *S*. *mansoni*. From multivariable analysis, increased odds of any STH infections were associated with not wearing shoes, adjusted odds ratio (aOR) = 1.36 (95%CI: 1.09–1.69); p = 0.007; high number of household members, aOR = 1.21 (95%CI: 1.04–1.41); p = 0.015; and school absenteeism of more than two days, aOR = 1.33 (95%CI: 1.01–1.80); p = 0.045. Further, children below five years had up to four times higher odds of getting STH infections, aOR = 4.68 (95%CI: 1.49–14.73); p = 0.008. However, no significant factors were identified for schistosomiasis, probably due to low prevalence levels affecting performance of statistical analysis.

**Conclusions:**

After five rounds of MDA, the program shows low prevalence of STH and schistosomiasis, however, not to a level where the infections are not a public health problem. With considerable inter-county infection prevalence heterogeneity, the program should adopt future MDA frequencies based on the county’s infection prevalence status. Further, the program should encourage interventions aimed at improving coverage among preschool age children and improving WASH practices as long-term infection control strategies.

## Author summary

This paper presents the findings of an evaluation survey conducted in Year 6 of the Kenya National School-Based Deworming (NSBD) Program, following five years (2012–2017) of prior baseline and subsequent impact monitoring. The survey was conducted in 20 counties, covering six regions in Kenya. The survey showed continued and considerable reductions in prevalence over time, with most marked decline for hookworm, followed by *Ascaris lumbricoides*. Overall, the mass drug administration (MDA) program has driven both STH and schistosomiasis prevalence to relatively low levels, however not to a point where they no longer constitute a public health problem in Kenya. For these neglected tropical diseases (NTDs), and STH in particular, there are relatively few published examples of programmatic impact assessments enabling refined decisions regarding helminth control strategies. Kenya’s experiences in implementing, monitoring, and evaluating a high-coverage NSBD program are a continuing and increasingly important success story for the country, which provides learnings of importance for the international community.

## Introduction

Soil-transmitted helminths (STH: three common species being *Ascaris lumbricoides*, the hookworms; *Necator americanus* and *Ancylostoma duodenale*, and *Trichuris trichiura)*, and schistosomiasis (mainly caused by *Schistosoma mansoni* and *S*. *haematobium*), are among the most common human parasitic infections globally. They affect the world’s most poorest and deprived communities especially in rural areas of Sub-Saharan Africa (SSA), Latin America, South East Asia and China [1,2]. These infections are mainly prevalent in warm and moist climates, strongly associated with poverty, where sanitation and hygiene are largely poor and drinking water is unsafe [3]. Current global estimates suggest that two billion people are infected with STH and a further four billion are at risk [4]. Over 200 million people are infected with schistosomiasis and over 700 million are at risk [2]; an estimated 90% of those infected live in SSA [4]. Children, mostly school age children (SAC: 5–14 years), in endemic areas bear the greatest burden of these infections due to their high susceptibility to frequent exposure to contaminated environment especially when playing, eating unwashed fruits or raw vegetables, and bathing in or drinking untreated water [5].

The infections are mainly caused by, for the case of STH infections, ingestion of eggs from the contaminated soil (*A*. *lumbricoides* and *T*. *trichiura*) or active penetration of the skin by larvae in the soil (hookworms) [5]. For the case of schistosomiasis, infection is usually caused by blood flukes (trematodes) with adult schistosomes invading the human blood vessels and the immune system where they excrete hundreds to thousands of eggs daily [6]. Preventive anthelminthic chemotherapy usually through mass drug administration (MDA) programs is the preferred and prioritized first line strategy by the World Health Organization (WHO) to overcome the burden and morbidity inflicted by these infections [5,7]. Additionally, interventions targeting improvement of access to water, sanitation and hygiene (WASH) are encouraged as a long-term and sustainable control measure [8]. Noting the goal in Kenya is aligned with the WHO’s goal of elimination as a public health problem, it is recognized that in order to make progress towards infection transmission interruption, activities incorporating MDA and possible expansion to MDA, socioeconomic improvements, increased WASH infrastructure, and modification of people’s behaviour aiming at reducing fecally contaminated environments through hygiene and health education, are considered to be crucial [8,9].

In Kenya, over five million SAC are estimated to be at risk of STH and schistosomiasis [10]. According to early geospatial mapping, these infections were determined to be mainly prevalent in the larger western parts of Kenya (Western and Nyanza regions), parts of South Rift Region, parts of the Coastal Region, with prevalence levels not warranting MDA (according to WHO decision trees [5,11]) in parts of Central, Eastern and North Eastern regions [12]. As such, the Government of Kenya, through the Ministry of Health (MoH) and Ministry of Education (MoE), launched a National School Based Deworming (NSBD) program in the year 2012 targeting the at-risk SAC living in 66 endemic sub-counties in 27 counties. The aim of the program was to reduce the infection prevalence (both STH and schistosomes) to a level where they no longer constituted a public health problem [13]. The impact of the NSBD program has been consistently monitored from 2012 to 2017 through a robust and extensive monitoring and evaluation (M&E) program led by the Kenya Medical Research Institute (KEMRI) using a series of cross-sectional surveys both pre- and post- MDA. The comprehensive design and the associated survey results of this M&E program have been published for baseline [13], three-year [14], and five-year [15] results.

Following the success of the first five years of monitoring, the M&E program embarked on an extensive evaluation of the country-wide status of these infections during Year 6 (the year 2018) of the NSBD program, with the view to inform the program’s next steps and if necessary re-categorize the country’s MDA requirement according to WHO guidelines for treatment strategies after five to six years of high MDA coverage [5]. This current study conducted surveys in 20 counties covering six regions (Nyanza, Western, Rift Valley, Coast, Eastern and North Eastern) with the objective of evaluating the parasitological outcomes in sites that had routine parasitological monitoring for five years (2012–2017: the sites included Nyanza, Western, Rift Valley and Coast regions), and those sites that had not had routine parasitological monitoring since baseline but had been participating in deworming (the sites included Eastern and North Eastern regions). Further, during this evaluation survey, the program collected individual, household and school level WASH and demographic characteristics in order to determine factors associated with the risk of STH and schistosomiasis in the national program in Kenya.

## Materials and methods

### Study design and sampling

The study utilized a cross-sectional design in a representative, stratified, two-stage sample of school children across six regions in Kenya. A sampling frame of all primary schools which were participating in the NSBD program within a county was taken. A sample size of 100 schools (five schools per county with highest STH prevalence) with approximately 108 children per school was calculated to be adequate to detect a 5% change in prevalence of STH infections, assuming power of 80% and test size of 5%, and considering the anticipated variance in prevalence. The schools were purposively selected based on Year 5 (the year 2017) STH infection endemicity prior to the study [15]. In each school, 18 children (nine girls and nine boys) were sampled randomly from each of the six classes; one early childhood development (ECD) class and classes two to six using random number tables, for a total of approximately 108 children per school.

### Survey procedures

The selected schools were visited three days prior to the survey date to explain the purpose of the survey to the school head teacher and the school committee; permission to conduct the study was sought at the school level. On the day of the survey, each selected child was given a container (poly pot) labeled with unique identifier and instructed to place a portion of his or her own stool sample in it. The stool samples were then processed in the laboratory within 24 hours and examined in duplicate for the presence of STH and *S*. *mansoni* eggs by two technicians using the Kato-Katz technique [16]. Additionally, urine samples were obtained only from children in the participating schools from Coastal, Eastern and North Eastern regions where *S*. *haematobium* is known to be focally prevalent. The urine samples were then processed using the urine filtration technique in the laboratory within 24 hours using polycarbonate membrane filters and examined in duplicate for the presence of *S*. *haematobium* eggs by two technicians [17]. However, children not present during the day of the survey were not included in the survey. As part of the NSBD program during MDA, all participating children were treated with albendazole (400 mg) and praziquantel (40 mg/kg) for STH and schistosomiasis respectively according to MoH and WHO guidelines [18].

### Data collection and management

Data was collected in two phases between 29th January to 17th February, 2018 and 8th to 24th May, 2018, approximately 12 months after the Year 5 MDA in each region. Single stool specimens were collected to assess prevalence and intensity of STH (*A*. *lumbricoides*, *T*. *trichiura*, and hookworm) and *S*. *mansoni* infections. Single urine specimens were additionally collected in Coast, Eastern and North Eastern regions to assess prevalence and intensity of *S*. *haematobium* infections. In all areas, pilot-tested survey questionnaires were administered to all the participating children to collect information on the participants’ demographic and their individual, household and school levels WASH related behaviours, practices and characteristics. Both survey questionnaires and laboratory reporting forms were programmed onto android-based smart phones and used to capture data electronically using the Open Data Kit (ODK) system which incorporated in-built data quality checks to reduce data entry errors [19].

### Ethics statement

Ethical approval for the study protocol was obtained from the Kenya Medical Research Institute (KEMRI)’s Scientific and Ethics Review Unit (SSC Number 2206). At county-level, approval was provided by the respective county health and education authorities. At school, parental consent was obtained based on passive opt-out consent rather than written opt-in consent due to the routine and low risk nature of the study procedure. Additionally, individual assent was obtained from each child before participation in the study. All data used were anonymised.

### Statistical analysis

Infection prevalence and average intensity of infection were calculated for STH and schistosomiasis and the 95% confidence intervals (CIs) determined using binomial and negative binomial regression models respectively, taking into account clustering by schools. Infection intensities were classified into light, moderate and heavy infections according to WHO guidelines (S1 Table) [20] and the prevalence of light, moderate and heavy infections together with 95%CIs obtained using a binomial regression model taking into account clustering by schools. We calculated the prevalence of each intensity class using two approaches; 1) when taking the denominator as the overall number of children examined, and 2) when taking the denominator as the total number of positive-children for each respective infection. The use of these two approaches enabled us to conveniently compare the morbidity due to these infections and for easy comparison to other studies.

WASH and sociodemographic conditions of interest from the questionnaires included reported individual, household and school-level variables that are known factors affecting STH or schistosomiasis prevalence. *Individual factors* included age, gender, handwashing, defecation and urination, soil-eating and shoe-wearing behaviours at school and home. *Household-level factors* included availability of toilet, anal cleansing material, handwashing facility equipped with water and soap, type of water source, as well as number of people living in an individual’s household. Information regarding type of household latrine (being improved or unimproved) was not collected as the surveys were conducted at school locations. *School-level factors* included interviewer-verified availability and type of school toilet facility, availability and type of handwashing facility equipped with water and soap, and availability of anal cleansing material at school. Additionally, latrine structural integrity and cleanliness were assessed by interviewers. Latrine structural integrity was assessed by the evidence of all the following: roof, walls with no holes, a functional lockable door, and a stable floor slab, while assessment of latrine cleanliness was determined by the absence of strong smell, absence of visible faeces on the latrine floor, and clean floor. Index scores for latrine structural integrity and cleanliness were created using factor analysis [21], with a score range of between zero and one, and with higher scores indicating greater cleanliness/structural integrity.

Overall, the WASH factors associated with STH or schistosomiasis prevalence were analyzed, first using univariable analysis and described as odds ratio (OR) using mixed effects logistic regression model at two levels; pupils nested within schools selected within counties. To select minimum adequate variables for multivariable analysis, an inclusion criterion of p-value <0.1 was pre-specified in a sequential (block-wise) variable selection method which selected covariates meeting the set criterion; however, sex and age were retained as fixed terms in the final models regardless of statistical significance due to their known importance. Adjusted OR (aOR), of the most parsimonious model, were obtained by mutually adjusting all minimum generated variables using multivariable mixed effects logistic regression model at 95%CI taking into account the hierarchical nature of the data.

All the statistical analyses were carried out using STATA version 14.1 (STATA Corporation, College Station, TX, USA). Graphs were developed using the *ggplot* package implemented in R [22]. School locations were mapped using ArcGIS Desktop version 10.2.2 software (Environmental Systems Research Institute Inc., Redlands, CA, USA).

## Results

Overall, 100 schools (9,801 children) with the median age of 10 years (range:1–21 years) were surveyed across 20 counties in Western, Nyanza, Rift Valley, Coast, Eastern and North Eastern regions prior to the Year 6 MDA. Five schools with a total of 108 children per school were surveyed from each county. Approximately half (50.2%) of the children were males. It is important to note that the wider age range here is due to inclusion of few younger children (<2 years) that were found in ECD classes, who properly might have accompanied their siblings or mothers to school on the day of the survey, on the other hand it is normal to get older children (≥15 years) in most primary schools in Kenya due to variety of reasons. Table 1 provides the number of schools and children examined by county as well as the range of school-level prevalence for both STH and schistosomiasis.

**Table 1:** Number of schools and children examined by county as well as school prevalence range (min—max) among school children in Kenya after five rounds of MDA.

County	Schools (children)	Median age (min-max)	School range STH prevalence (min-max)	School range *S*. *mansoni* prevalence (min-max)	School range *S*. *haematobium* prevalence (min-max)
Bomet	5 (541)	10.0 (3–18)	13.0–39.8	0–0	_ns
Bungoma	5 (519)	9.5 (4–15)	0.9–9.3	0–0	_ns
Busia	5 (540)	11.0 (4–17)	0.9–56.5	0–41.7	_ns
Garissa	5 (197)	10.0 (4–14)	0–0	0–0	0–3.4
Homa Bay	5 (535)	10.0 (5–15)	16.7–35.2	0–15.3	_ns
Kakamega	5 (539)	10.0 (5–16)	17.6–38.9	0–31.5	_ns
Kericho	5 (540)	9.0 (1–16)	13.0–22.2	0–0	_ns
Kilifi	5 (507)	10.0 (4–17)	1.0–19.3	0–0	0–0.9
Kisii	5 (532)	9.0 (4–16)	9.3–37.0	0–0	_ns
Kisumu	5 (540)	10.0 (5–16)	0–8.3	0–13.9	_ns
Kitui	5 (540)	10.0 (2–18)	0–0.9	0–2.8	0–0
Kwale	5 (522)	9.0 (4–16)	0.9–11.1	0–1.9	0–5.7
Makueni	5 (522)	10.0 (4–14)	0–1.9	0–13.9	0–0
Wajir	5 (112)	9.5 (3–14)	0–0	0–0	0–0
Migori	5 (539)	10.0 (4–19)	0.9–4.6	0–0	_ns
Mombasa	5 (526)	10.0 (4–16)	0–5.6	0–0.9	0–0.9
Narok	5 (516)	10.0 (4–21)	12.0–33.7	0–0	_ns
Nyamira	5 (511)	10.0 (3–14)	0–39.8	0–0	_ns
Taita Taveta	5 (491)	9.0 (3–15)	0–0.9	0–0.9	0–0
Vihiga	5 (532)	10.0 (1–14)	12.1–49.5	0–1.9	_ns
**Total**	**100 (9,801)**	**10.0 (1–21)**	**0–56.5**	**0–41.7**	**0–5.7**

**_ns** Indicates counties which were not surveyed for *S*. *haematobium*

### STH infections

The overall prevalence of any STH infection was 12.9% (95%CI: 10.4–16.1) with species-specific prevalence of 9.7% (95%CI: 7.5–12.6) for *A*. *lumbricoides*, 3.6% (95%CI: 2.2–5.8) for *T*. *trichiura* and 1.0% (95%CI: 0.6–1.5) for hookworm. Similarly, the overall mean intensity were highest for *A*. *lumbricoides*, 741 epg (95%CI: 535–1027), followed by *T*. *trichiura*, 15 epg (95%CI: 8–27), and then hookworm, 6 epg (95%CI: 2–16) (Table 2).

**Table 2:** Overall prevalence % (95%CI), mean intensity epg (95%CI) of infections and relative reductions (RR) % (p-value) among school children in Kenya after five rounds of MDA.

Survey	No. schools (children) surveyed	STH combined	Hookworm	*A*. *lumbricoides*	*T*. *trichiura*	*S*. *mansoni*	*S*. *haematobium*
**Prevalence, % (95%CI)**							
Year 1 Baseline*	199 (21,432)	32.3 (30.0–34.8)	15.4 (13.6–17.6)	18.1 (15.8–20.7)	6.7 (5.4–8.2)	2.4 (1.5–4.1)	18.0 (13.0–24.9)
Year 3 *	199 (21,011)	16.4 (14.4–18.6)	2.3 (1.8–3.0)	11.9 (10.2–13.9)	4.5 (3.4–6.0)	1.7 (0.8–3.6)	7.9 (3.8–16.2)
Year 5 *	198 (20,941)	13.5 (11.6–15.7)	1.3 (1.0–1.6)	9.6 (8.0–11.5)	4.1 (3.1–5.5)	2.0 (1.2–3.2)	3.9 (1.7–9.0)
Year 6 Evaluation^$^	100 (9,801)	12.9 (10.4–16.1)	1.0 (0.6–1.5)	9.7 (7.5–12.6)	3.6 (2.2–5.8)	2.2 (1.2–4.3)	0.3 (0.1–1.0)
RR (Y1Baseline–Y6Evaluation)	-	61.7 (p<0.001)	93.6 (p<0.001)	52.9 (p<0.001)	42.7 (p = 0.006)	7.9 (p = 0.779)	98.5 (p<0.001)
**Average Intensity, epg (95%CI)**							
Year 1 Baseline*	199 (21,432)	-	63 (50–81)	1659 (1378–1998)	33 (11–105)	14 (5–41)	20 (11–39)
Year 3 *	199 (21,011)	-	8 (5–14)	960 (801–1151)	17 (11–26)	6 (2–16)	7 (3–16)
Year 5 *	198 (20,941)	-	10 (5–19)	917 (750–1121)	16 (10–26)	5 (3–10)	4 (1–12)
Year 6 Evaluation^$^	100 (9,801)	-	6 (2–16)	741 (535–1027)	15 (8–27)	12 (5–31)	0 (0–1)
RR (Y1Baseline–Y6Evaluation)	-	-	90.7 (p<0.001)	61.1 (p<0.001)	58.3 (p = 0.201)	13.4 (p = 0821)	99.3 (p<0.001)

* Indicates surveys done under Year 1 (Y1), Year 3 (Y3) and Year 5 (Y5) monitoring and evaluation and included 200 schools in four regions **[13–15]**

$ Indicates surveys done under this current assessment and included 100 schools in six regions

Overall, the undifferentiated STH prevalence reduced by 61.7% (p<0.001) from the baseline of 32.3%; similarly, specific species indicated significant declines over the period. Hookworm reduced by 93.6% (p<0.001) from a baseline prevalence of 15.4%, *A*. *lumbricoides* reduced by 52.9% (p<0.001) from baseline prevalence of 18.1% and *T*. *trichiura* reduced by 42.7% (p<0.001) from initial prevalence of 6.7%. Similar declines were observed for mean intensity of infections (Table 2).

Analysis of undifferentiated STH prevalence by demographics (sex and age group) showed that there was no significant difference between male and female children (*χ*^2^ = 0.31, p = 0.578) or by age groups (*χ*^2^ = 4.08, p = 0.130). Whilst non-significant slightly higher hookworm infection prevalence was seen in older children (<5 years: 0%, 5–14 years: 1.0% and >14 years: 1.2%), analyzing differences at this low level of prevalence is affected by the limits of diagnostic accuracy. For *A*. *lumbricoides*, non-significantly higher infection prevalence was seen in younger children (<5 years: 14.7%, 5–14 years: 9.7% and >14 years: 4.9%) and for *T*. *trichiura*, non-significantly higher infection prevalence was observed among those aged 5 to 14 years at 3.6%, compared to those aged <5 years or >14 years (Table 3).

**Table 3:** STH and schistosomiasis prevalence % (95%CI) by demographics among school children in Kenya after five rounds of MDA.

Demographics	N (%)	STH combined	Hookworm	*A*. *lumbricoides*	*T*. *trichiura*	*S*. *mansoni*	*S*. *haematobium*
Gender							
Male	4,814 (50.2%)	13.1 (10.5–16.3)	1.1 (0.1–1.8)	9.9 (7.6–12.9)	3.6 (2.2–5.8)	2.3 (1.2–4.6)	0.3 (0.1–1.5)
Female	4,771 (49.8%)	12.7 (10.1–15.9)	0.8 (0.5–1.3)	9.6 (7.4–12.5)	3.5 (2.2–5.7)	2.2 (1.1–4.2)	0.2 (0.1–0.8)
Difference in prevalence (Chi-square *χ*^2^)	-	*χ*^2^ = 0.31, p = 0.578	*χ*^2^ = 2.30, p = 0.130	*χ*^2^ = 0.22, p = 0.638	*χ*^2^ = 0.01, p = 0.939	*χ*^2^ = 0.17, p = 0.678	*χ*^2^ = 0.11, p = 0.739
Age group							
< 5	95 (1.0%)	15.8 (9.5–26.1)	0	14.7 (8.6–25.2)	1.1 (0.1–7.8)	0	0
5–14	9,408 (98.1%)	12.9 (10.4–16.0)	1.0 (0.7–1.5)	9.7 (7.5–12.6)	3.6 (2.2–5.8)	2.3 (1.2–4.3)	0.3 (0.1–1.0)
> 14	82 (0.9%)	6.1 (2.4–15.7)	1.2 (0.2–9.0)	4.9 (2.0–12.0)	1.2 (0.2–8.1)	3.7 (0.9–14.8)	0
Difference in prevalence (Chi-square *χ*^2^)	-	*χ*^2^ = 4.08, p = 0.130	*χ*^2^ = 1.00, p = 0.607	*χ*^2^ = 4.90, p = 0.086	*χ*^2^ = 3.08, p = 0.215	*χ*^2^ = 2.93, p = 0.231	*χ*^2^ = 0.15, p = 0.930

**Fig 1:**
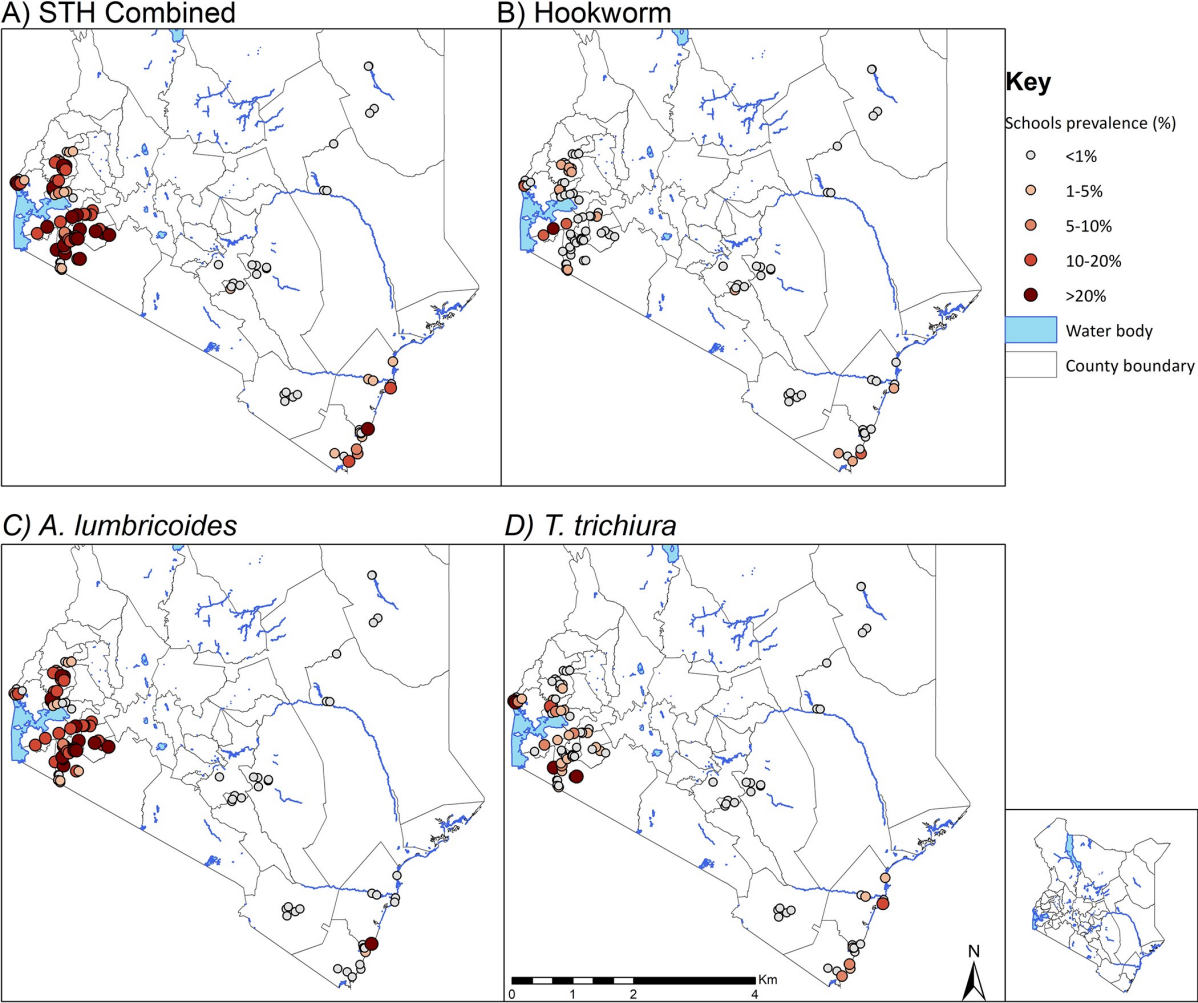
STH Prevalence distribution after five years of deworming in Kenya.

Fig 1 shows considerable heterogeneity in the prevalence of STH across the surveyed counties after five years of MDA. Some schools, mainly in the western part of Kenya, still had prevalence of any STH above 20%. All surveyed schools in Eastern and North Eastern regions had STH prevalence below 1%.

There was no infection among surveyed schools in Garissa and Wajir counties. Three counties (Kitui, Makueni and Taita Taveta) had STH prevalence below 1%, six counties (Bungoma, Kilifi, Kisumu, Kwale, Migori, and Mombasa) had STH prevalence between 1% and 10%, one county (Kericho) had STH prevalence between 10% and 20%, and the remaining eight counties had STH prevalence between 20% and 50% (Table 4). County-specific mean intensities of infection are shown in Table 5.

**Table 4:** Overall and county prevalence % (95%CI) for STH and schistosomiasis among school children in Kenya after five rounds of MDA.

County	No. children (%) N = 9,801	STH combined	Hookworm	*A*. *lumbricoides*	*T*. *trichiura*	*S*. *mansoni*	*S*. *haematobium*
Bomet	541 (5.5%)	24.0 (16.5–34.8)	0	23.4 (15.8–34.6)	1.1 (0.3–4.1)	0	_ns
Bungoma	519 (5.3%)	5.1 (2.5–10.4)	0.2 (0–1.4)	4.9 (2.5–9.8)	0	0	_ns
Busia	540 (5.5%)	23.4 (9.1–6.0)	1.1 (0.2–7.9)	4.8 (2.0–11.6)	21.2 (7.4–60.5)	17.6 (6.7–46.0)	_ns
Garissa	197 (2.0%)	0	0	0	0	0	0.5 (0.1–4.8)
Homa Bay	535 (5.5%)	23.0 (17.4–30.4)	5.6 (2.7–11.6)	16.5 (10.3–26.4)	4.9 (2.6–9.1)	4.9 (1.4–16.6)	_ns
Kakamega	539 (5.5%)	23.9 (17.3–33.2)	2.8 (2.3–3.4)	22.8 (15.9–32.6)	1.3 (0.9–1.9)	6.7 (1.1–41.6)	_ns
Kericho	540 (5.5%)	16.9 (13.8–20.9)	0.4 (0.1–2.7)	16.0 (13.1–19.6)	1.3 (0.6–2.7)	0	_ns
Kilifi	507 (5.2%)	4.8 (1.4–16.6)	0.6 (0.1–2.4)	0.4 (0.1–1.3)	4.0 (0.9–17.1)	0	0.2 (0–1.4)
Kisii	532 (5.4%)	21.6 (13.8–33.9)	0.4 (0.1–1.3)	20.8 (13.3–32.7)	1.5 (0.6–4.1)	0	_ns
Kisumu	540 (5.5%)	3.2 (1.3–7.6)	0.2 (0–1.3)	1.1 (0.6–2.1)	2.0 (0.8–5.5)	4.6 (1.5–13.9)	_ns
Kitui	540 (5.5%)	0.4 (0.1–1.2)	0.4 (0.1–1.2)	0	0	1.1 (0.3–3.7)	0
Kwale	522 (5.3%)	6.3 (3.5–11.3)	2.8 (1.3–5.8)	0	3.9 (1.4–11.3)	0.4 (0.1–2.8)	1.1 (0.2–8.1)
Makueni	522 (5.3%)	0.6 (0.2–2.1)	0.6 (0.2–2.1)	0	0	3.7 (0.9–15.8)	0
Wajir	112 (1.1%)	0	0	0	0	0	0
Migori	539 (5.5%)	2.4 (1.4–4.3)	0.7 (0.3–1.9)	1.3 (0.6–3.0)	0.6 (0.2–2.1)	0	_ns
Mombasa	526 (5.4%)	2.2 (0.8–6.0)	0.8 (0.1–5.5)	0.4 (0–3.3)	1.0 (0.3–3.3)	0.2 (0–1.4)	1.9 (0–1.3)
Narok	516 (5.3%)	24.5 (17.4–34.5)	0	12.4 (6.1–25.3)	14.4 (7.3–28.1)	0	_ns
Nyamira	511 (5.2%)	22.9 (13.3–39.3)	0.4 (0.1–2.9)	22.1 (13.1–37.1)	0.8 (0.2–3.4)	0	_ns
Taita Taveta	491 (5.0%)	0.2 (0–1.4)	0	0.2 (0–1.4)	0	0.4 (0.1–1.3)	0
Vihiga	532 (5.4%)	30.7 (19.2–49.1)	0.9 (0.5–1.8)	30.3 (18.7–49.0)	7.8 (3.5–17.0)	0.4 (0.1–2.7)	_ns
**Total**	**9,801 (100%)**	**12.9 (10.4–16.1)**	**1.0 (0.6–1.5)**	**9.7 (7.5–12.6)**	**3.6 (2.2–5.8)**	**2.2 (1.2–4.3)**	**0.3 (0.1–1.0)**

**_ns** Indicates counties which were not surveyed for *S*. *haematobium*

**Table 5:** Overall and county mean intensity epg (95%CI) for STH and schistosomiasis among school children in Kenya after five rounds of MDA.

County	No. children (%) N = 9,801	Hookworm	*A*. *lumbricoides*	*T*. *trichiura*	*S*. *mansoni*	*S*. *haematobium*
Bomet	541 (5.5%)	0	1353 (901–2033)	3 (1–11)	0	_ns
Bungoma	519 (5.3%)	0	131 (60–286)	0	0	_ns
Busia	540 (5.5%)	1 (0–6)	296 (113–777)	87 (28–269)	142 (50–397)	_ns
Garissa	197 (2.0%)	0	0	0	0	0
Homa Bay	535 (5.5%)	41 (7–251)	749 (487–1152)	3 (2–7)	6 (1–31)	_ns
Kakamega	539 (5.5%)	4 (3–6)	1924 (1415–2617)	1 (0–3)	53 (8–368)	_ns
Kericho	540 (5.5%)	1 (0–4)	911 (695–1194)	1 (1–3)	0	_ns
Kilifi	507 (5.2%)	0 (0–1)	0	43 (8–239)	0	0 (0–1)
Kisii	532 (5.4%)	1 (0–7)	1864 (851–4086)	2 (0–5)	0	_ns
Kisumu	540 (5.5%)	0 (0–3)	155 (92–263)	1 (0–3)	5 (1–21)	_ns
Kitui	540 (5.5%)	0	0	0	2 (0–5)	0
Kwale	522 (5.3%)	11 (5–26)	0	15 (7–35)	3 (0–19)	1 (0–6)
Makueni	522 (5.3%)	1 (0–3)	0	0	6 (1–31)	0
Wajir	112 (1.1%)	0	0	0	0	0
Migori	539 (5.5%)	2 (1–6)	16 (4–60)	14 (2–93)	0	_ns
Mombasa	526 (5.4%)	1 (0–8)	303 (37–2459)	3 (1–8)	0 (0–1)	0
Narok	516 (5.3%)	0	789 (259–2404)	78 (30–201)	0	_ns
Nyamira	511 (5.2%)	42 (6–305)	1610 (787–3295)	9 (1–62)	0	_ns
Taita Taveta	491 (5.0%)	0	29 (4–200)	0	2 (0–8)	0
Vihiga	532 (5.4%)	2 (1–4)	3422 (1909–6133)	17 (7–42)	0 (0–3)	_ns
**Total**	**9,801 (100%)**	**6 (2–16)**	**741 (535–1027)**	**15 (8–27)**	**12 (5–31)**	**0 (0–1)**

**_ns** Indicates counties which were not surveyed for *S*. *haematobium*

At regional level, STH infections were generally more prevalent in Rift Valley Region (21.8%) with species-specific prevalence of 17.4% for *A*. *lumbricoides*, 5.5% for *T*. *trichiura* and 0.1% for hookworm, followed by Western Region (20.9%) with species-specific prevalence of 15.8% for *A*. *lumbricoides*, 7.7% for *T*. *trichiura* and 1.3% for hookworm. Low percentages of infections were observed in Eastern Region where only hookworm was present at 0.5% (noting that diagnostic accuracy of Kato Katz at this level of prevalence is questionable). However, no STH infection was observed in any of the surveyed schools of North Eastern Region.

Using both approaches, we found that STH infections were predominantly of light intensity, followed by moderate and then heavy intensity. In particular, using the first approach, the prevalence of light infections were 5.8% (n = 561) for *A*. *lumbricoides*, 5.2% (n = 322) for *T*. *trichiura* and 2.8% (n = 91) for hookworm, and prevalence of heavy infections were observed for *A*. *lumbricoides* only at 1.9% (n = 3). The prevalence of moderate to heavy infections for any STH infections was 6.0% (n = 399), with the majority of the moderate to heavy infections being for *A*. *lumbricoides*. The first approach showed that the prevalence of moderate to heavy intensity of the STH infections has significantly reduced since baseline for all the STH infections except that of *T*. *trichiura* (Table 6).

**Table 6:** Prevalence % (95%CI) and year 1 (Y1) to year 6 (Y6) relative reductions (RR) % (p-value) of light, moderate and heavy intensity of infections among school children in Kenya after five rounds of MDA.

Infections	Total children examined (Total positive)	Light infections^#^			Moderate infections^#^			Heavy infections^#^			Moderate-heavy infections^#^		
		n	Calculated using total children examined as denominator*	Calculated using total positives as denominator**	n	Calculated using total children examined as denominator*	Calculated using total positives as denominator**	n	Calculated using total children examined as denominator*	Calculated using total positives as denominator**	n	Calculated using total children examined as denominator*	Calculated using total positives as denominator**
**STH infections:**													
STH combined													
Y1 baseline	21,432 (6,274)	4,435	23.7 (22.0–25.5)	70.7 (67.3–74.2)	1,808	9.8 (8.3–11.4)	28.8 (25.6–32.5	31	0.2 (0.1–0.5)	0.5 (0.2–1.5)	1,839	9.9 (8.5–11.6)	29.3 (26.1–33.0)
Y6 evaluation	9,801 (1,274)	843	8.6 (6.9–10.8)	59.1 (51.3–68.0)	392	4.0 (2.9–5.4)	27.5 (22.0–34.3)	7	2.0 (0.9–4.2)	0.5 (0.2–1.1)	399	6.0 (4.4–8.2)	28.0 (22.4–34.9)
RR% (p-value)	-		63.9% (p<0.001)	16.4% (p = 0.014)		58.8% (p<0.001)	4.7% (p = 0.651)		*Increased* (11.8%, p<0.001)	0.7% (p = 0.992)		39.6% (p<0.001)	4.6% (p = 0.653)
*A*. *lumbricoides*													
Y1 baseline	21,432 (3,843)	2,086	11.2 (10.0–12.7)	54.3 (51.1–57.7)	1,757	9.5 (8.0–11.2)	45.7 (42.5–49.2)	**-**	-^$^	-^$^	1,757	9.5 (8.0–11.2)	45.7 (42.5–49.2)
Y6 evaluation	9,801 (935)	561	5.8 (4.5–7.5)	60.0 (54.4–66.2)	371	3.9 (2.8–5.3)	39.7 (34.3–45.9)	3	1.9 (0.9–4.2)	0.3 (0.1–1.0)	374	5.7 (4.1–7.9)	40.0 (34.5–46.3)
RR% (p-value)	-		47.9% (p<0.001)	*Increased* (10.5%, p = 0.062)		59.1% (p<0.001)	13.2% (p = 0.065)		-^$^	-^$^		39.2% (p = 0.002)	12.5% (p = 0.082)
Hookworm													
Y1 baseline	21,432 (2,856)	2,809	14.9 (13.0–17.1)	98.4 (95.6–99.7)	33	0.2 (0.1–0.3)	1.2 (0.7–1.8)	14	0.1 (0–0.1)	0.5 (0.3–0.9)	47	0.3 (0.2–0.4)	1.6 (1.1–2.4)
Y6 evaluation	9,801 (94)	91	2.8 (1.6–4.8)	96.8 (92.3–98.2)	0	0	0	3	0 (0–0.1)	3.2 (0.7–13.6)	3	0 (0–0.1)	3.2 (0.7–13.6)
RR% (p-value)	-		81.3% (p<0.001)	1.6% (p = 0.412)		100% (p<0.001)	100% (p<0.001)		58.5% (p = 0.279)	*Increased* (551.1% (p = 0.019)		87.6% (p = 0.007)	*Increased* (93.4% (p = 0.387)
*T*. *trichiura*													
Y1 baseline	21,432 (1,169)	1,112	6.0 (4.8–7.6)	95.1 (92.0–98.4	40	0.2 (0.1–0.3)	3.4 (2.4–4.8)	17	0.1 (0–0.7)	1.5 (0.2–10.1)	57	0.3 (0.2–0.6)	4.9 (2.5–9.4)
Y6 evaluation	9,801 (346)	322	5.2 (3.5–7.8)	93.1 (90.4–95.8)	23	0.2 (0.1–0.5)	6.6 (4.4–10.1)	**1**	0 (0–0.1)	0.3 (0–2.2)	24	0.2 (0.1–0.5)	6.9 (4.7–10.3)
RR% (p-value)	-		13.4% (p = 0.458)	2.2% (p = 0.337)		*Increased* (11.4%, p = 0.770)	*Increased* (94.3%, p = 0.012)		88.6% (p = 0.125)	80.1% (p = 0.253)		18.4% (p = 0.674)	*Increased* (42.3%, p = 0.367)
**Schistosome infections:**													
Any schistosome													
Y1 baseline	21,432 (701)	351	91.9 (88.4–95.6)	50.1 (38.1–65.8)	130	0.7 (0.4–1.4)	28.9 (21.1–39.6)	219	1.2 (0.6–2.2)	31.2 (21.6–45.2)	132	1.9 (1.1–3.3)	2.1 (1.3–3.5)
Y6 evaluation	9,801 (223)	91	63.7 (55.0–73.7)	40.8 (31.2–53.3)	74	0.8 (0.4–1.6)	34.6 (29.6–40.4)	58	2.5 (1.3–4.7)	26.0 (17.6–38.5)	26	3.2 (1.9–5.6)	1.8 (0.3–9.8)
RR% (p-value)	-		30.7% (p<0.001)	18.5% (p = 0.175)		*Increased* (8.2%, p = 0.819)	*Increased* (19.7%, p = 0.276)		*Increased* (109.9%, p = 0.077)	16.7% (p = 0.412)		*Increased* (72.1%, p = 0.108)	13.4% (p = 0.847)
*S*. *mansoni*													
Y1 baseline	21,432 (450)	183	1.0 (0.7–1.5)	40.7 (27.2–60.9)	130	0.7 (0.4–1.4)	28.9 (21.1–39.6)	137	0.7 (0.3–1.8)	30.4 (17.3–53.5)	267	1.4 (0.7–2.9)	59.3 (45.0–78.2)
Y6 evaluation	9,801 (214)	85	0.9 (0.5–1.5)	39.7 (30.2–52.2)	74	0.8 (0.4–1.6)	34.6 (29.6–40.4)	55	2.5 (1.3–4.6)	25.7 (16.9–39.0)	129	3.2 (1.8–5.6)	60.3 (50.4–72.1)
RR% (p-value)	-		10.0% (p = 0.716)	2.3% (p = 0.904)		*Increased* (10.3%, p = 0.776)	*Increased* (19.7%, p = 0.276)		*Increased* (232.9%, p = 0.021)	15.6% (p = 0.558)		*Increased* (123.5%, p = 0.038)	*Increased* (1.6%, p = 0.905)
*S*. *haematobium*													
Y1 baseline	1,399 (252)	169	93.4 (90.0–96.9)	67.1 (56.8–79.2)	**-**	-^$^	-^$^	83	5.9 (3.5–10.1)	32.9 (23.5–46.3)	83	5.9 (3.5–10.1)	32.9 (23.5–46.3)
Y6 evaluation	3,417 (9)	6	65.2 (56.3–75.5)	66.7 (49.0–90.7)	**-**	-^$^	-^$^	3	0.1 (0–0.4)	33.3 (18.0–61.7)	3	0.1 (0–0.4)	33.3 (18.0–61.7)
RR% (p-value)	-		30.2% (p<0.001)	0.6% (p = 0.974)	**-**	-^$^	-^$^		98.5% (p<0.001)	*Increased* (1.2%, p = 0.973)		98.5% (p<0.001)	*Increased* (1.2%, p = 0.973)

-$ Indicate that prevalence of intensity was not assessed at that particular cut-off point

n; indicates the number positive for each intensity class

**#** Prevalence of each intensity class was calculated using two approaches

1)* when taking the denominator as the overall number of children examined, and

2)** when taking the denominator as the total number of positive-children for each infection. The use of these two approaches enabled us to conveniently compare the morbidity due to these infections and for easy comparison to other studies.

### Schistosome infections

The overall prevalence of *S*. *mansoni* infection was 2.2% (95%CI: 1.2–4.3) and for *S*. *haematobium*, 0.3% (95%CI: 0.1–1.0). Respective mean intensities of infection of 12 epg (95%CI: 5–31) and 0 eggs/mL (95%CI: 0–1) were seen (Table 2).

Prevalence of schistosomiasis overall was very low, and at these levels only *S*. *haematobium* prevalence reduced significantly, by 98.5% (p<0.001) from baseline prevalence of 18.0%. *S*. *mansoni* showed a non-significant reduction of 7.9% (p = 0.779) from initial prevalence of 2.4%. However, it is difficult to assess significant reductions in both species of *Schistosoma* due to poor diagnostic performance of Kato-Katz at low levels of prevalence, and also because the baseline prevalence of *S*. *mansoni* was very low to commence with. Similar decline patterns were observed for mean intensities of infection (Table 2).

Analysis of schistosomiasis prevalence by demographics (sex and age group) showed no significant difference between male and female children in *S*. *mansoni* infection prevalence (*χ*^2^ = 0.17, p = 0.678), or in *S*. *haematobium* prevalence (*χ*^2^ = 0.11, p = 0.739). Similarly, there was no significant difference in *S*. *mansoni* (*χ*^2^ = 2.93, p = 0.231) and *S*. *haematobium* (*χ*^2^ = 0.15, p = 0.930) prevalence by age group (Table 3).

**Fig 2:**
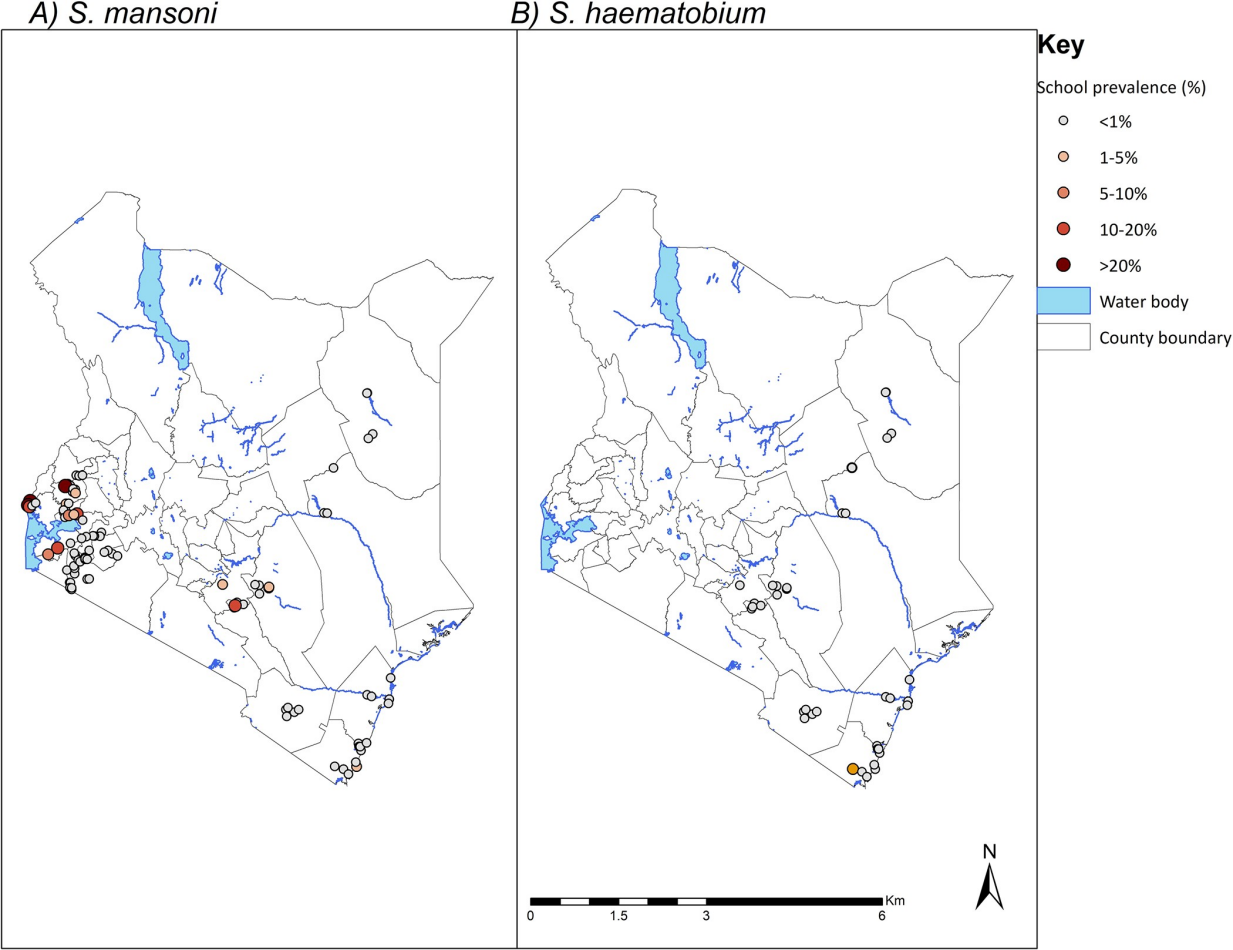
Schistosomiasis prevalence distribution after five years of deworming in Kenya.

Fig 2 provides the schistosomiasis prevalence distribution in all the surveyed counties after five rounds of MDA. Nearly all the surveyed schools had both *S*. *mansoni* and *S*. *haematobium* prevalence below 1%. However, a pocket of schools, mainly in the western part of Kenya, had prevalence of *S*. *mansoni* above 10%. There was no *S*. *mansoni* infection in the surveyed schools in ten counties, below 1% prevalence in four counties, between 1% and 10% in five counties and above 10% only in Busia County. Similarly, *S*. *haematobium* prevalence was zero in Makueni, Wajir and Taita Taveta counties, below 1% in Garissa and Kilifi counties, and between 1% and 10% in Kwale and Mombasa counties (Table 4). County-specific mean intensities of infection are shown in Table 5.

At regional level, *S*. *mansoni* infection was most prevalent in Western (6.3%), followed by Eastern (2.4%), Nyanza (1.9%) and Coast regions (0.3%), however no *S*. *mansoni* infection was observed in North Eastern and Rift Valley regions. Additionally, *S*. *haematobium* infection was only observed in Coast (0.4%) and North Eastern (0.3%) at very low levels, with no observed infection in Eastern Region.

Similarly, using both approaches, we found that intensity of schistosomiasis were predominantly of light intensity, except that of *S*. *mansoni* which were predominantly of heavy infections according to first approach. In particular, using the first approach, the prevalence of light infections were 0.9% (n = 85) and 65.2% (n = 6), and prevalence of heavy infections were 2.5% (n = 55) and 0.1% (n = 3) respectively for *S*. *mansoni* and *S*. *haematobium*. The prevalence of moderate to heavy infections for any schistosome was 3.2% (n = 26) with the majority of the moderate to heavy infections being for *S*. *mansoni*. The first approach showed that the prevalence of moderate to heavy intensity of *S*. *haematobium* significantly reduced since baseline (RR = 98.5%, p<0.001) but it instead increased by more than two-folds for *S*. *mansoni* (Table 6).

### Individual, household and school WASH characteristics

All the 9,801 children surveyed from 100 schools were administered with a questionnaire, where they reported on their WASH practices and behaviours both at school and at home. However, we would like to point out that some of the indictors measured like number of household occupants and shoe-waering behaviour were proxy indicators of poverty but not risk factors *per se*. Table 7 gives the WASH characteristics, overall and stratified by region.

**Table 7:** Pupil, household and school WASH characteristics overall and stratified by regions in Kenya after five rounds of MDA.

Characteristics	Overall (N = 9,801) n (%) or mean (SD)	Coast (n = 2,046) n (%) or mean (SD)	Nyanza (n = 2,657) n (%) or mean (SD)	Western (n = 2,130) n (%) or mean (SD)	Rift Valley (n = 1,597) n (%) or mean (SD)	Eastern (n = 1,062) n (%) or mean (SD)	N. Eastern (n = 309) n (%) or mean (SD)
***STH infections***							
Any STH infection prevalence	1,242 (12.9%)	68 (3.4%)	381 (14.5%)	442 (20.9%)	346 (21.8%)	5 (0.5%)	0
Hookworm prevalence	94 (1.0%)	21 (1.1%)	39 (1.5%)	27 (1.3%)	2 (0.1%)	5 (0.5%)	0
Hookworm mean intensity^$^	6	3	17	2	0	0	0
*A*. *lumbricoides* prevalence	935 (9.7%)	5 (0.3%)	321 (12.2%)	333 (15.8)	276 (17.4%)	0	0
*A*. *lumbricoides* mean intensity^$^	741	83	865	1451	1021	0	0
*T*. *trichiura* prevalence	346 (3.6%)	45 (2.3%)	52 (2.0%)	162 (7.7%)	87 (5.5%)	0	0
*T*. *trichiura* mean intensity^$^	15	15	6	27	27	0	0
***Schistosome infections***							
*S*. *mansoni* prevalence	214 (2.2%)	5 (0.3%)	51 (1.9%)	133 (6.3%)	0	25 (2.4%)	0
*S*. *mansoni* mean intensity^$^	12	1	2	50	0	4	0
*S*. *haematobium* prevalence	9 (0.3%)	8 (0.4%)	-	-	-	0	1 (0.3%)
*S*. *haematobium* mean intensity^$^	0	0	-	-	-	0	0
**Individual and household characteristics**							
Boys	4,771 (49.8%)	987 (49.9%)	1,318 (50.5%)	1,054 (50.6%)	776 (49.8%)	518 (49.3%)	161 (53.7%)
Age	9.6 (2.4)	9.5 (2.2)	9.4 (2.5)	9.7 (2.3)	9.6 (2.5)	9.9 (2.3)	9.4 (2.1)
Number of household occupants	6.8 (2.6)	6.8 (2.5)	6.6 (3.0)	6.8 (2.0)	7.2 (2.8)	6.6 (2.1)	6.6 (1.9)
Wearing shoes	8,101 (84.5%)	1,516 (76.6%)	2,523 (96.6%)	1,400 (67.2%)	1,530 (98.2%)	857 (81.5%)	275 (91.7%)
Soil-eating behaviour	2,596 (27.1%)	78 (3.9%)	1,121 (42.9%)	982 (47.1%)	72 (4.6%)	332 (31.6%)	11 (3.7%)
Improved water source*	4,866 (49.7%)	1,857 (90.8%)	907 (34.1%)	601 (28.2%)	763 (47.8%)	540 (50.9%)	198 (64.1%)
Toilet/latrine available	9,329 (97.3%)	1,907 (96.3%)	2,497 (95.6%)	2,070 (99.3%)	1,548 (99.4%)	1,012 (96.3%)	295 (98.3%)
Handwashing facility with soap and water always available	1,422 (14.5%)	229 (11.2%)	748 (28.2%)	65 (3.1%)	361 (22.6%)	18 (1.7%)	1 (0.3%)
Tissue/water for anal cleansing always available	5,174 (54.0%)	1,217 (61.5%)	1,596 (61.1%)	510 (24.5%)	1,498 (96.2%)	288 (27.4%)	65 (21.7%)
**School characteristics**	**Overall (n = 100)**	**Coast (n = 20)**	**Nyanza (n = 25)**	**Western (n = 20)**	**Rift valley (n = 15)**	**Eastern (n = 10)**	**N. Eastern (n = 10)**
Number of children in school	526.7 (316.8)	645.7 (446.4)	482.6 (260.6)	758.7 (247.4)	359.3 (154.5)	326.4 (186.1)	386.3 (176.8)
Improved water source*	40 (40.0%)	9 (45.0%)	6 (24.0%)	7 (35.0%)	9 (60.0%)	3 (30.0%)	6 (60.0%)
Latrine/toilet available	99 (99.0%)	19 (95.0%)	25 (100%)	20 (100%)	15 (100%)	10 (100%)	10 (100%)
Pupils per latrine [median (IQR)]	54.9 (94.2)	54.9 (100)	54.4 (93.2)	98.4 (128.9)	37.3 (32.8)	37.8 (35.5)	113.9 (128.7)
Handwashing facility with soap and water always available	3 (3.0%)	0	3 (12.0%)	0	0	0	0
Drinking water always available	36 (48.0%)	0	14 (56.0%)	4 (26.7%)	11 (73.3)	5 (50.0%)	2 (20.0%)
Number of months without water in the school	4.6 (5.9)	4.0 (2.6)	2.3 (2.3)	3.9 (4.2)	3.7 (3.3)	6.2 (4.3)	12.4 (14.3)
Tissue/water for anal cleansing always available	10 (10.2%)	14 (14.0%)	15 (14.8%)	9 (8.9%)	3 (3.1%)	4 (4.2%)	12 (11.7%)

$ Infection intensity was measured using egg per gram and displayed as arithmetic mean of two readings

* Improved water source was defined as access to tap water, boreholes, protected wells or springs, and rain water

The overall reported average number of household occupants was 6.8 people (standard deviation (SD) = 2.6 people). At the time of the interview, the majority 8,101 (84.5%) of the pupils were wearing shoes. Geophagy was not uncommon at 2,596 (27.1%) of the pupils. Nearly half 4,866 (49.7%) of the pupils reported use of an improved water source for drinking at their household. Reported latrine coverage (any type of latrine) at household level was high 9,329 (97.3%), however, fewer pupils reported always having a handwashing facility equipped with water and soap 1,422 (14.5%), or tissues/water for anal cleansing 5,174 (54.0%) available in their households.

School WASH conditions varied considerably by region as well as by county (Table 7). The average number of pupils per school was 527 (SD = 377). Improved water sources were interviewer-observed in 40 (40.0%) of the schools. Nearly all of the schools had at least one latrine block 99 (99.0%). However, only 45 (45.5%) of the latrines were of ventilated improved pit (VIP) latrine type. Few schools had a handwashing facility equipped with water and soap 3 (3.0%), or tissue/water always available for anal cleansing 10 (10.2%). The average pupil per latrine ratio was 55:1 with only 27 (27.0%) of the schools meeting the Government of Kenya (GoK) standard of 30 male pupils per latrine, and 25 female pupils per latrine (with separate latrines for males and females). Additionally, on the day of the visit a number of latrines were visually observed by interviewers to have excessive smell (95 out of 292 latrines; 32.5%), visible feces outside or around (41 out of 292 latrines; 14.0%), or excessive flies (72 out of 292 latrines; 24.7%). Good cleanliness (118 out of 292 latrines; 40.3%), and good structural integrity (251 out of 292 latrines; 86.0%) were reported for the majority of the latrines.

**Fig 3:**
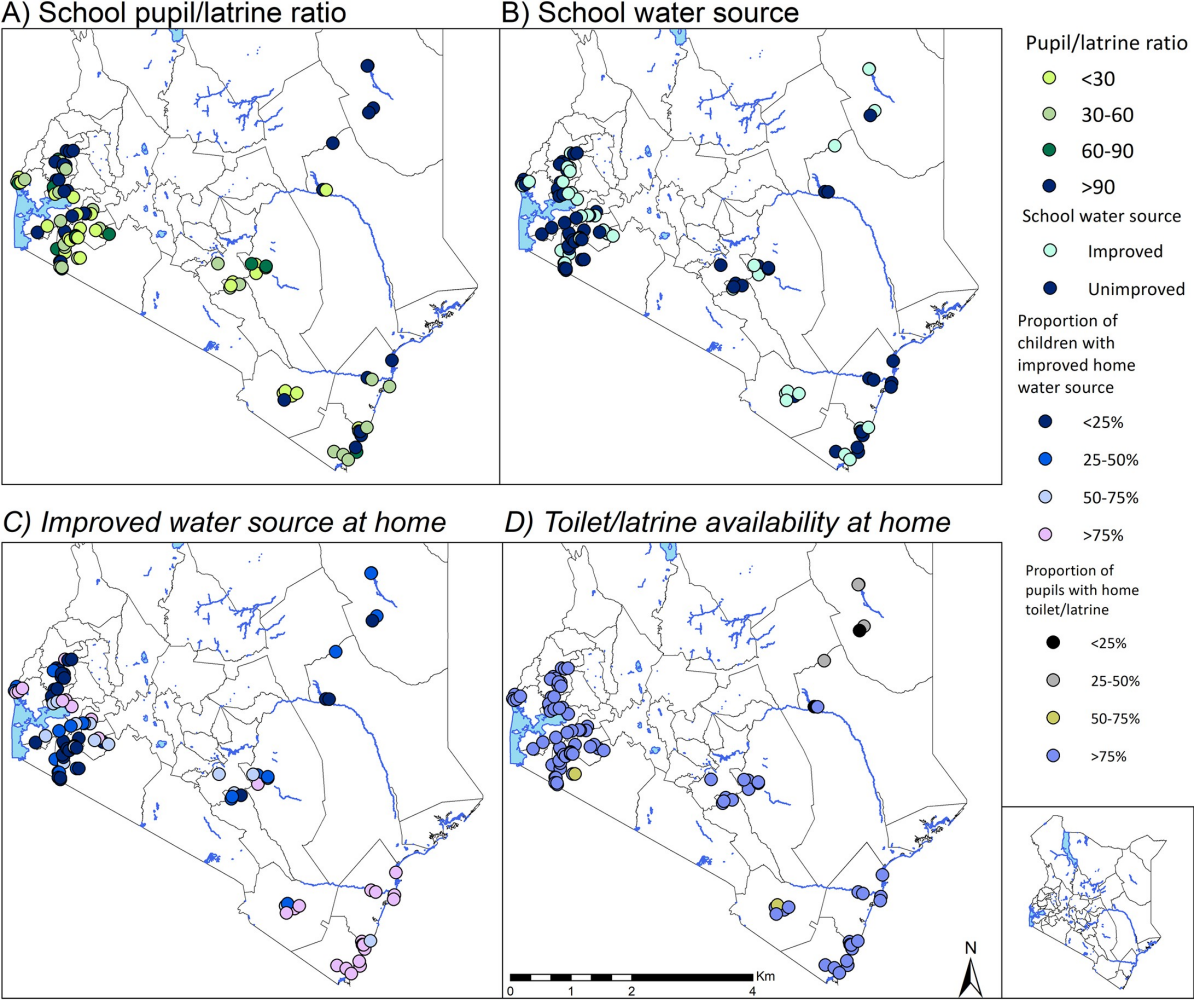
Distribution of school and household water and sanitation conditions in Kenya.

Fig 3 provides the distribution of school and household water and sanitation infrastructure. Higher number of pupils to latrine (i.e. >90 pupils/latrine) was observed in all schools in the North Eastern Region and in several schools in Western and Coastal regions. Most schools across all the regions were visually observed to have an unimproved water source. Less than 25% of children in western part of Kenya reported low availability of improved water source at home. Reported latrine availability at household level was good (latrine coverage was above 75%) among all the interviewed pupils in all the regions.

### Univariable and multivariable analysis of factors associated with STH infections

A large number of individual, household and school level WASH and socioeconomic factors were assessed in a univariable model and revealed significant associations with STH infections as shown in Table 8. They were then fitted in a multivariable model after adjusting for other factors, however, many of them did not remain significant (Table 9). Not wearing shoes on day of interview (adjusted odds ratio (aOR) = 1.36, p = 0.007), and household membership of more than five members per household (aOR = 1.21, p = 0.015), showed significant association with increased odds of any STH infections. Additionally, a gradient effect relative to never being absent from school was evident; although one day’s absence was non-significant (aOR = 1.05, p = 0.612), two days’ absence (aOR = 1.32, p = 0.029), and more than two days’ absence (aOR = 1.33, p = 0.045) were both significantly associated with increased odds of STH infection. Children aged 5–14 years (aOR = 3.25, p = 0.015), relative to those aged over 14 years, were shown to have three times greater odds of STH infections; on the other hand, children aged less than 5 years had over four times greater odds of STH infection than those aged over 14 years (aOR = 4.68, p = 0.008). Availability of tissue/newspaper/water for anal cleansing at home (aOR = 0.77, p = 0.005), and households possessing electricity (aOR = 0.75, p = 0.001) were associated with lower odds of any STH infections. Sex was not significantly associated with STH infection.

**Table 8:** Univariable associations between WASH conditions and STH infections among school children in Kenya after five rounds of MDA.

Factors	STH combined (n = 1,242)		*A*. *lumbricoides* (n = 935)		Hookworm (n = 94)		*T*. *trichiura* (n = 346)	
	OR (95%CI)	p-value	OR (95%CI)	p-value	OR (95%CI)	p-value	OR (95%CI)	p-value
**Individual factors:**								
Male children	1.07 (0.94–1.22)	0.314	1.05 (0.90–1.22)	0.534	1.43 (0.94–2.17)	0.098*	1.08 (0.85–1.38)	0.518
ECD children	1.68 (1.43–1.99)	<0.001**	1.86 (1.55–2.23)	<0.001**	0.89 (0.50–1.59)	0.691	1.28 (0.94–1.74)	0.121
Soil-eating behavior	1.16 (1.01–1.32)	0.030**	1.67 (1.45–1.93)	<0.001**	0.64 (0.38–1.07)	0.088*	0.41 (0.30–0.56)	<0.001**
Not wearing shoes	1.42 (1.14–1.77)	0.002**	1.53 (1.18–1.97)	0.001**	1.45 (0.80–2.61)	0.219	1.18 (0.81–1.73)	0.390
Age group								
< 5 years vs > 14 years	4.57 (1.46–14.2)	0.009**	3.78 (1.09–13.1)	0.036**	Insufficient obs		1.92 (0.11–35.5)	0.662
5–14 years vs > 14 years	3.21 (1.24–8.32)	0.016**	1.85 (0.65–5.29)	0.252	0.90 (0.12–6.87)	0.918	9.41 (1.22–72.4)	0.031**
Did not report receiving treatment during last MDA	1.20 (0.93–1.53)	0.159	1.29 (0.97–1.71)	0.080*	1.07 (0.53–2.15)	0.856	0.88 (0.55–1.41)	0.599
**Household factors:**								
*Household members*:								
More than 5 members vs 1–5 members	1.21 (1.04–1.41)	0.016**	1.24 (1.04–1.47)	0.014**	1.37 (0.82–2.29)	0.224	1.16 (0.87–1.56)	0.321
*Household head level of education*:								
No formal education vs Secondary and above	1.23 (1.00–1.52)	0.054*	1.45 (1.15–1.83)	0.002**	0.58 (0.29–1.16)	0.121	0.91 (0.59–1.40)	0.656
Primary education vs Secondary and above	1.28 (1.07–1.54)	0.008**	1.32 (1.07–1.62)	0.009**	0.85 (0.50–1.46)	0.566	1.05 (0.72–1.53)	0.797
*Roof materials*:								
Iron sheets vs tiles	0.68 0.34–1.40	0.297	1.03 (0.40–2.68)	0.948	0.66 (0.12–3.55)	0.627	0.56 (0.18–1.75)	0.318
Grass/thatch/makuti vs tiles	0.72 0.35–1.49	0.375	1.10 (0.42–2.94)	0.842	0.50 (0.09–2.93)	0.444	0.59 (0.19–1.88)	0.374
*Floor materials*:								
Wooden vs cement/tiles	1.05 (0.36–3.04)	0.924	0.99 (0.31–3.13)	0.981	Insufficient obs		0.81 (0.08–7.84)	0.855
Earth/sand vs cement/tiles	1.21 (1.03–1.43)	0.023**	1.19 (0.99–1.44)	0.061*	1.32 (0.77–2.27)	0.318	1.33 (0.96–1.85)	0.090*
*Wall materials*:								
Clay/mud vs stone/bricks/cement	1.21 (1.00–1.47)	0.055*	1.19 (0.95–1.49)	0.130	1.50 (0.79–2.83)	0.212	1.27 (0.89–1.82)	0.183
Wood vs stone/bricks/cement	1.15 (0.75–1.75)	0.518	1.13 (0.73–1.76)	0.587	Insufficient obs		0.94 (0.29–3.02)	0.914
Iron sheets vs stone/bricks/cement	0.99 (0.65–1.51)	0.952	1.07 (0.68–1.69)	0.782	1.03 (0.25–4.17)	0.971	0.88 (0.34–2.28)	0.794
*Household possessions*:								
Radio	0.91 (0.77–1.09)	0.297	0.83 (0.68–1.02)	0.073*	0.85 (0.51–1.41)	0.533	1.08 (0.78–1.49)	0.649
Television	0.73 (0.61–0.88)	0.001**	0.64 (0.52–0.80)	<0.001**	0.65 (0.36–1.17)	0.151	0.88 (0.64–1.20)	0.421
Mobile phone	0.96 (0.71–1.29)	0.784	0.94 (0.66–1.33)	0.716	0.67 (0.31–1.42)	0.292	0.96 (0.60–1.53)	0.864
Sofa set	0.84 (0.71–1.00)	0.047**	0.85 (0.70–1.04)	0.110	0.84 (0.50–1.42)	0.516	0.83 (0.61–1.14)	0.252
Bicycle	1.03 (0.88–1.21)	0.701	1.02 (0.86–1.23)	0.791	1.58 (1.00–2.50)	0.048**	1.14 (0.86–1.50)	0.365
Motorcycle	0.90 (0.74–1.10)	0.294	0.88 (0.70–1.11)	0.281	1.00 (0.59–1.71)	0.993	0.90 (0.64–1.27)	0.548
Electricity	0.72 (0.61–0.86)	<0.001**	0.68 (0.56–0.84)	<0.001**	0.74 (0.43–1.27)	0.277	0.96 (0.71–1.31)	0.807
Car	0.76 (0.50–1.18)	0.220	0.71 (0.42–1.18)	0.184	1.73 (0.66–4.54)	0.262	0.60 (0.26–1.38)	0.232
Toilet/latrine available	1.42 (0.84–2.38)	0.189	1.00 (0.53–1.87)	0.999	2.66 (0.80–8.85)	0.111	2.22 (0.77–6.41)	0.140
Share toilet/latrine with other households	1.04 (0.88–1.23)	0.670	1.11 (0.91–1.34)	0.297	0.89 (0.54–1.46)	0.635	0.75 (0.54–1.03)	0.072*
Used toilet/latrine to defecate last time at home	1.08 (0.77–1.51)	0.645	0.96 (0.67–1.39)	0.836	3.80 (0.91–15.8)	0.067*	0.97 (0.51–1.88)	0.937
Tissue/newspaper/water always available for anal cleansing	0.74 (0.62–0.88)	0.001**	0.73 (0.60–0.89)	0.001**	0.91 (0.53–1.56)	0.734	0.82 (0.56–1.21)	0.319
Handwashing facility with soap and water always available	0.84 (0.67–1.05)	0.126	0.87 (0.68–1.12)	0.284	1.14 (0.59–2.22)	0.693	0.70 (0.45–1.08)	0.107
Improved water source	1.12 (0.93–1.33)	0.227	1.11 (0.91–1.35)	0.318	1.62 (0.96–2.75)	0.072*	1.06 (0.77–1.45)	0.715
**School factors:**								
Handwashing facility with soap and water always available	0.66 (0.25–1.75)	0.408	0.68 (0.23–2.03)	0.493	Insufficient obs		0.68 (0.09–5.32)	0.711
Tissue/newspaper/water always available for anal cleansing	0.96 (0.82–1.12)	0.604	0.96 (0.81–1.14)	0.645	0.82 (0.51–1.34)	0.438	0.84 (0.63–1.12)	0.230
Drinking water always available	0.86 (0.68–1.08)	0.197	0.85 (0.65–1.11)	0.240	1.09 (0.61–1.94)	0.775	1.02 (0.68–1.52)	0.928
Always use school latrine/toilet	0.67 (0.35–1.28)	0.224	0.90 (0.43–1.88)	0.778	Insufficient obs		0.26 (0.09–0.73)	0.010**
*Days absent from school in the last one week*:								
One day vs never absent	1.05 (0.88–1.25)	0.590	1.07 (0.88–1.31)	0.500	0.96 (0.54–1.70)	0.886	1.14 (0.83–1.56)	0.434
Two days vs never absent	1.35 (1.05–1.73)	0.019**	1.43 (1.09–1.89)	0.010**	0.90 (0.40–2.03)	0.808	1.67 (1.06–2.63)	0.027**
More than two days vs never absent	1.36 (1.02–1.82)	0.035**	1.38 (1.00–1.92)	0.053*	1.42 (0.69–2.92)	0.342	1.61 (0.97–2.66)	0.064*

* Indicates p-value <0.1, used to determine inclusion criteria in multivariable models

**Indicates p-value <0.05, which determine a significant association

**Table 9:** Multivariable associations between WASH conditions and STH combined infections among school children in Kenya after five rounds of MDA.

Factors	STH combined (n = 1,242)	
	aOR (95%CI)	p-value
**Individual and household factors**		
Male children	1.05 (0.92–1.20)	0.461
Not wearing shoes on day of interview	1.36 (1.09–1.69)	0.007**
Age group		
< 5 years vs > 14 years	4.68 (1.49–14.73)	0.008**
5–14 years vs > 14 years	3.25 (1.25–8.46)	0.015**
*Household members*:		
More than 5 members vs 1–5 members	1.21 (1.04–1.41)	0.015**
Tissue/newspaper/water always available for anal cleansing	0.77 (0.65–0.92)	0.005**
*Household possessions*:		
Electricity	0.75 (0.62–0.89)	0.001**
**School factors**		
*Days absent from school in the last one week*:		
One day vs never absent	1.05 (0.88–1.25)	0.612
Two days vs never absent	1.32 (1.03–1.70)	0.029**
More than two days vs never absent	1.33 (1.01–1.80)	0.045**

** Indicates significant associations (p<0.05)

Nearly all the above factors; not wearing shoes (aOR = 1.46, p = 0.003), high household membership (aOR = 1.24, p = 0.001) and school absenteeism of two days (aOR = 1.40, p = 0.016), and more than two days (aOR = 1.35, p = 0.072) similarly showed higher significant risk for *A*. *lumbricoides* infection. The availability of tissue/newspaper/water for anal cleansing at home (aOR = 0.78, p = 0.015) and availability of television at home (aOR = 0.68, p = 0.001) showed significant association with lower odds of *A*. *lumbricoides* infection (Table 10). Children aged less than 5 years had significantly greater odds of infection than children aged over 14 years (aOR = 3.56, p = 0.045), but children aged 5–14 years did not (aOR = 1.72, p = 0.308). Sex was not significantly associated with *A*. *lumbricoides* infection.

**Table 10:** Multivariable associations between WASH conditions and *Ascaris lumbricoides* infection among school children in Kenya after five rounds of MDA.

Factors	*A*. *lumbricoides* (n = 935)	
	aOR (95%CI)	p-value
**Individual and household factors**		
Male children	1.03 (0.88–1.19)	0.740
Not wearing shoes	1.46 (1.13–1.89)	0.003**
Age group		
< 5 years vs > 14 years	3.56 (1.03–12.35)	0.045**
5–14 years vs > 14 years	1.72 (0.60–4.92)	0.308
*Household members*:		
More than 5 members vs 1–5 members	1.24 (1.04–1.47)	0.001**
Tissue/newspaper/water always available for anal cleansing	0.78 (0.64–0.95)	0.015**
*Household possessions*:		
Television	0.68 (0.54–0.85)	0.001**
**School factors**		
*Days absent from school in the last one week*:		
One day vs never absent	1.07 (0.88–1.31)	0.482
Two days vs never absent	1.40 (1.06–1.85)	0.016**
More than two days vs never absent	1.35 (0.97–1.88)	0.072

** Indicates significant associations (p<0.05)

For *T*. *trichiura* infection, children aged 5–14 years (aOR = 10.01, p = 0.027) and two days’ school absenteeism (aOR = 1.72, p = 0.020) were the only risk factors identified; though more than two days’ absenteeism was mildly non-significantly associated with increased odds of infection, (aOR = 1.59, p = 0.072). Always using school latrine (aOR = 0.26, p = 0.010) was shown as a protective factor (Table 11). A multivariable model for hookworm infection was not run due to insufficient observations.

**Table 11:** Multivariable associations between WASH conditions and *Trichuris trichiura* infection among school children in Kenya after five rounds of MDA.

Factors	*T*. *trichiura* (n = 346)	
	aOR (95%CI)	p-value
**Individual and household factors**		
Male children	1.10 (0.86–1.41)	0.439
Age group		
< 5 years vs > 14 years	2.12 (0.11–39.22)	0.614
5–14 years vs > 14 years	10.01 (1.30–77.08)	0.027**
**School factors**		
Always use school latrine/toilet	0.26 (0.09–0.72)	0.010**
*Days absent from school in the last one week*:		
One day vs never absent	1.16 (0.84–1.59)	0.372
Two days vs never absent	1.72 (1.09–2.71)	0.020**
More than two days vs never absent	1.59 (0.96–2.63)	0.072

** Indicates significant associations (p<0.05)

### Univariable and multivariable analysis of factors associated with schistosomiasis

Univariable analysis of individual, household and school level WASH and socioeconomic factors showed no significant associations between prevalence of schistosomiasis and any of the variables of interest as shown in Table 12. However, factors such as not wearing shoes (odds ratio (OR) = 1.20, p = 0.501), not receiving treatment during the last MDA (OR = 1.08, p = 0.779), sharing toilet/latrine with other households (OR = 1.12, p = 0.515), and school absenteeism (OR = 1.04, p = 0.873) showed increased odds of *S*. *mansoni* infection, though not significant. Additionally, only school absenteeism (OR = 1.13, p = 0.894) showed non-significant increased odds of *S*. *haematobium* infection. As such, and also due to low number of observations, multivariable analysis for factors associated with schistosomiasis prevalence was not conducted.

**Table 12:** Univariable associations between WASH conditions and schistosomiasis among school children in Kenya after five rounds of MDA.

Factors	*S*. *mansoni* (n = 214)		*S*. *haematobium* (n = 9)	
	OR (95%CI)	p-value	OR (95%CI)	p-value
**Individual factors:**				
Male children	1.11 (0.82–6.31)	0.501	1.33 (0.34–5.18)	0.680
ECD children	0.77 (0.49–1.20)	0.248	0.01 (0–0.01)	0.878
Soil-eating behavior	0.29 (0.18–0.46)	<0.001**	Insufficient obs	
Not wearing shoes	1.20 (0.79–1.82)	0.396	1.00 (0.22–4.66)	0.996
Age group				
< 5 years vs > 14 years	0.01 (0–0.01)	0.828	0.14 (0.02–0.19)	0.996
5–14 years vs > 14 years	1.77 (0.48–6.49)	0.389	Insufficient obs	
Did not receive treatment during last MDA	1.08 (0.64–1.83)	0.779	0.01 (0–0.01)	0.893
**Household factors:**				
*Household members*:				
More than 5 members vs 1–5 members	1.00 (0.71–1.41)	0.993	0.43 (0.10–1.90)	0.264
*Household head level of education*:				
No formal education vs Secondary and above	0.92 (0.57–1.49)	0.735	0.52 (0.07–4.22)	0.543
Primary education vs Secondary and above	0.83 (0.51–1.34)	0.446	0.26 (0.05–1.31)	0.103
*Roof materials*:				
Iron sheets vs tiles	0.14 (0.01–1.60)	0.114	0.14 (0–14.52)	0.404
Grass/thatch/makuti vs tiles	0.13 (0.01–1.57)	0.109	0.14 (0–18.20)	0.428
*Floor materials*:				
Wooden vs cement/tiles	1.06 (0.08–13.29)	0.965	Insufficient obs	
Earth/sand vs cement/tiles	0.81 (0.57–1.15)	0.240	0.76 (0.08–7.21)	0.813
*Wall materials*:				
Clay/mud vs stone/bricks/cement	0.68 (0.46–1.00)	0.050*	0.71 (0.08–6.40)	0.762
Wood vs stone/bricks/cement	0.01 (0–0.01)	0.900	Insufficient obs	
Iron sheets vs stone/bricks/cement	0.85 (0.40–1.81)	0.671	Insufficient obs	
*Household possessions*:				
Radio	0.97 (0.67–1.39)	0.851	5.45 (0.23–128.83)	0.294
Television	0.95 (0.66–1.37)	0.774	5.58 (0.44–71.44)	0.186
Mobile phone	0.90 (0.53–1.53)	0.697	0.69 (0.07–6.97)	0.755
Sofa set	1.06 (0.75–1.49)	0.753	2.47 (0.46–13.24)	0.291
Bicycle	1.39 (1.00–1.92)	0.050*	0.59 (0.13–2.61)	0.488
Motorcycle	0.97 (0.65–1.47)	0.902	0.98 (0.19–5.00)	0.980
Electricity	1.24 (0.87–1.76)	0.230	9.06 (0.48–171.55)	0.142
Car	0.99 (0.42–2.32)	0.981	0.01 (0–0.01)	0.958
Toilet/latrine available	0.59 (0.29–1.23)	0.161	Insufficient obs	
Share toilet/latrine with other households	1.12 (0.79–1.60)	0.515	0.29 (0.02–3.65)	0.339
Used toilet/latrine to defecate last time at home	1.04 (0.47–2.32)	0.922	Insufficient obs	
Tissue/newspaper/water always available for anal cleansing	0.84 (0.54–1.31)	0.445	Insufficient obs	
Handwashing facility with soap and water always available	0.86 (0.51–1.45)	0.565	0.01 (0–0.01)	0.948
Improved water source	0.91 (0.64–1.30)	0.609	Insufficient obs	
**School factors:**				
Handwashing facility with soap and water always available	0.01 (0–0.01)	0.926	0.01 (0–0.01)	0.962
Tissue/newspaper/water always available for anal cleansing	0.98 (0.69–1.40)	0.940	0.54 (0.02–15.60)	0.720
Drinking water always available	0.75 (0.39–1.42)	0.373	Insufficient obs	
Always use school latrine/toilet	1.11 (0.11–11.01)	0.928	Insufficient obs	
*Days absent from school in the last one week*:				
One day vs never absent	1.04 (0.67–1.60)	0.873	0.31 (0.03–2.90)	0.303
Two days vs never absent	0.73 (0.29–1.78)	0.483	0.59 (0.06–5.52)	0.645
More than two days vs never absent	0.40 (0.12–1.33)	0.134	1.13 (0.19–6.69)	0.894

* Indicates p-value <0.1, used to determine inclusion criteria in multivariable models

**Indicates p-value <0.05, which determine a significant association

## Discussion

Since Kenya has implemented the NSBD program for five years, it is important to conduct an evaluation of the program with the view to refine MDA requirements in line with the WHO guidelines [5]. Additionally, this evaluation enables us to understand the variation of the program impact between and within counties as well as risk factors associated with infection.

### STH and schistosome infections

From this evaluation, STH infections have declined tremendously after five rounds of treatment to prevalence of 12.9%; a highly significant reduction of 61.7% since baseline. Less marked but also important, are reductions in schistosomiasis prevalence to 2.2% (*S*. *mansoni*) and 0.3% (*S*. *haematobium*). Mean intensities for these parasites similarly reduced over this period. These results indicate that Kenya, via its NSBD, is making good headway towards achieving elimination of these diseases as a public health problem. WHO recommendations indicate that where STH five-year evaluation prevalence is assessed at 10–20%, preventive chemotherapy should continue once per year, and where schistosomiasis five-year evaluation prevalence is assessed at 1–10% it should continue once after every two years [5].

Whilst drops in each STH species prevalence were significant, the species indicated different decline rates perhaps reflecting their different dynamic mechanisms and reactions to albendazole [23,24]. *A*. *lumbricoides* has been previously shown to have high levels of re-infections [14], while single-dose oral albendazole, as is given in STH control programs, has been shown to be less efficacious against *T*. *trichiura* [25]. For schistosomiasis, whilst only the decline in *S*. *haematobium* was significant, numbers overall for both schistosome species were very low. The non-significant decline in *S*. *mansoni* might be attributed to several factors such as the poor sensitivity of the Kato-Katz diagnostic technique at low prevalence, its already low baseline prevalence [26], along with a known gap in treatment coverage as there was a lack of availability of praziquantel in one year of the NSBD program. These factors might have resulted in the observed high prevalence of heavy-intensity of any schistosomiasis especiacially *S*. *mansoni* infection and as such this prevalence is still above the newly-defined WHO target of <1% [27], indicating that schistosomiasis in the country is still a public health problem, and there is no question that praziquantel treatments should continue. WHO guidelines further provide for conducting of serological surveys where five-year follow-up prevalence of schistosomiasis is <1%, before making any decision to stop MDA [5]. Further, it is concerning that a few pocket of schools mainly in the western part of Kenya had high *S*. *mansoni* prevalence above 10%, this could be influenced by their nearness to Lake Victoria and hence indication of high schistosomiasis transmission around that lake and its associated islands, a finding that has been supported by various studies [28–30], and calls for critical need for targeted control of schistosomiasis.

**Fig 4:**
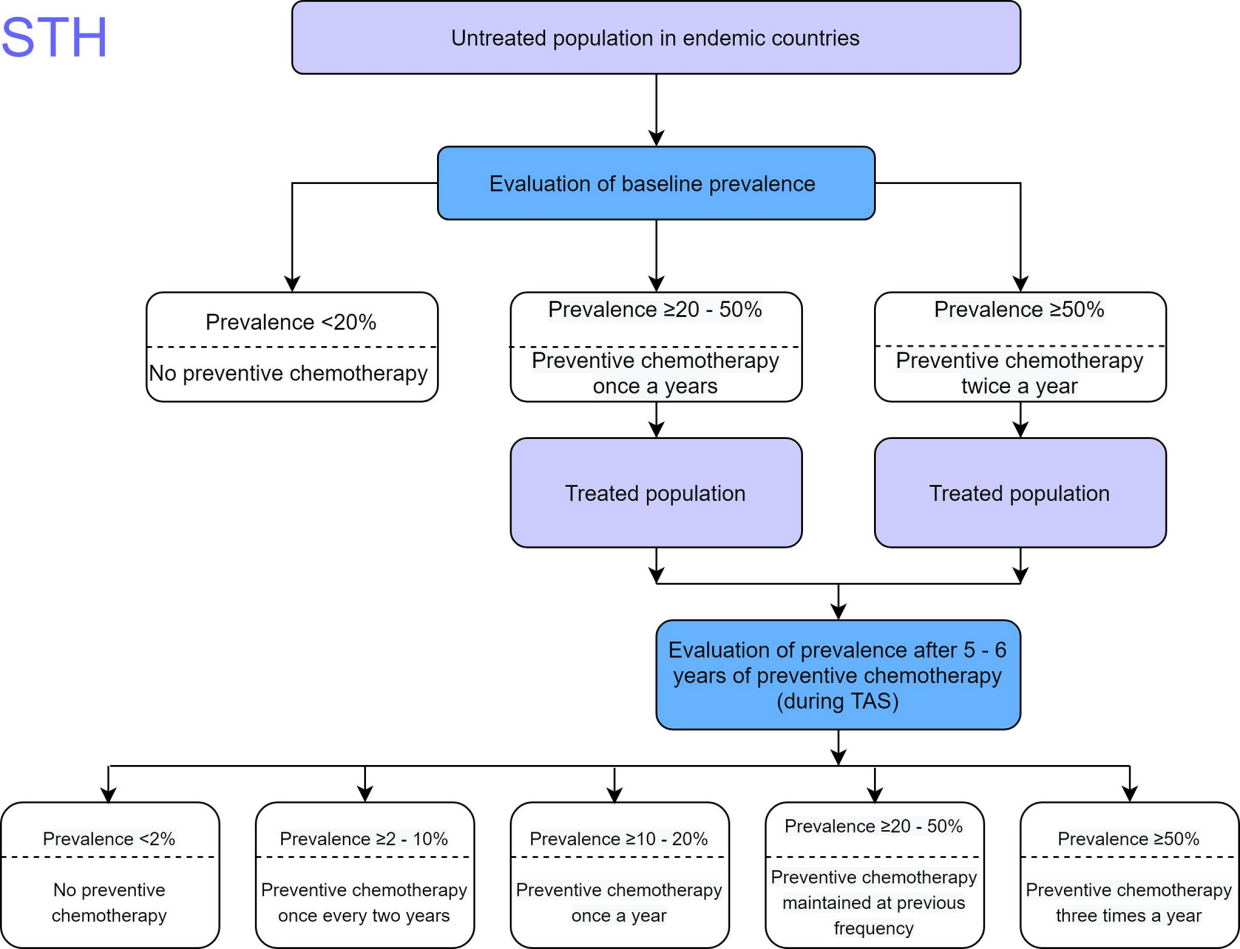
WHO guidelines decision tree for STH infections after five to six years of deworming.

Examples of large-scale helminth programs achieving levels of reduction warranting reducing or ceasing MDA are still relatively unusual, and whilst WHO decision trees provide guidance on reduction and cessation thresholds, it is also generally acknowledged that MDA needs to continue for some time after achieving the definition of elimination as a public health problem to prevent resurgence of infection. Whilst as noted above the national program has not achieved this level yet, with infection heterogeneity within counties, some but not all counties potentially meet definitions of elimination of STH or schistosomiasis as a public health problem. As the survey was not powered to county level, this is a picture that is emerging that warrants careful investigation going forward. This evaluation did not find any STH infection among the schools surveyed in Garissa and Wajir counties, either a feature of the non-random selection of schools or possibly indicating no ongoing biological transmission of STH in those counties and probably in the greater North Eastern Region of Kenya. This is in agreement with past empirical and research studies in the region, which have indicated that there could be little or no active biological transmission of these infections mainly due to the harsh arid climate in the region [31]. Future surveys should encompass random (for STH) and a mixture of both random and purposive (for schistosomiasis) selection of sites to further verify these results. However, considering the results, Kitui and Makueni counties in the Eastern Region and Taita Taveta County in the upper part of the Coast Region, which all had STH prevalence below 1%, could benefit from development and implementation of surveillance strategies including careful monitoring of coverage and compliance, until there is further evidence that planning to stop MDA would not be followed by resurgence of infection. Six counties, Bungoma, Kisumu, Migori, Kilifi, Kwale and Mombasa with STH prevalence between 1 to <10%, and Kericho County with prevalence between 10 to <20% would need to continue MDA once every year. Eight counties with prevalence between 20 to <50% would be required to maintain the previous MDA pattern (which is also once every year) under the current treatment guidelines (Fig 4, [11]).

**Fig 5:**
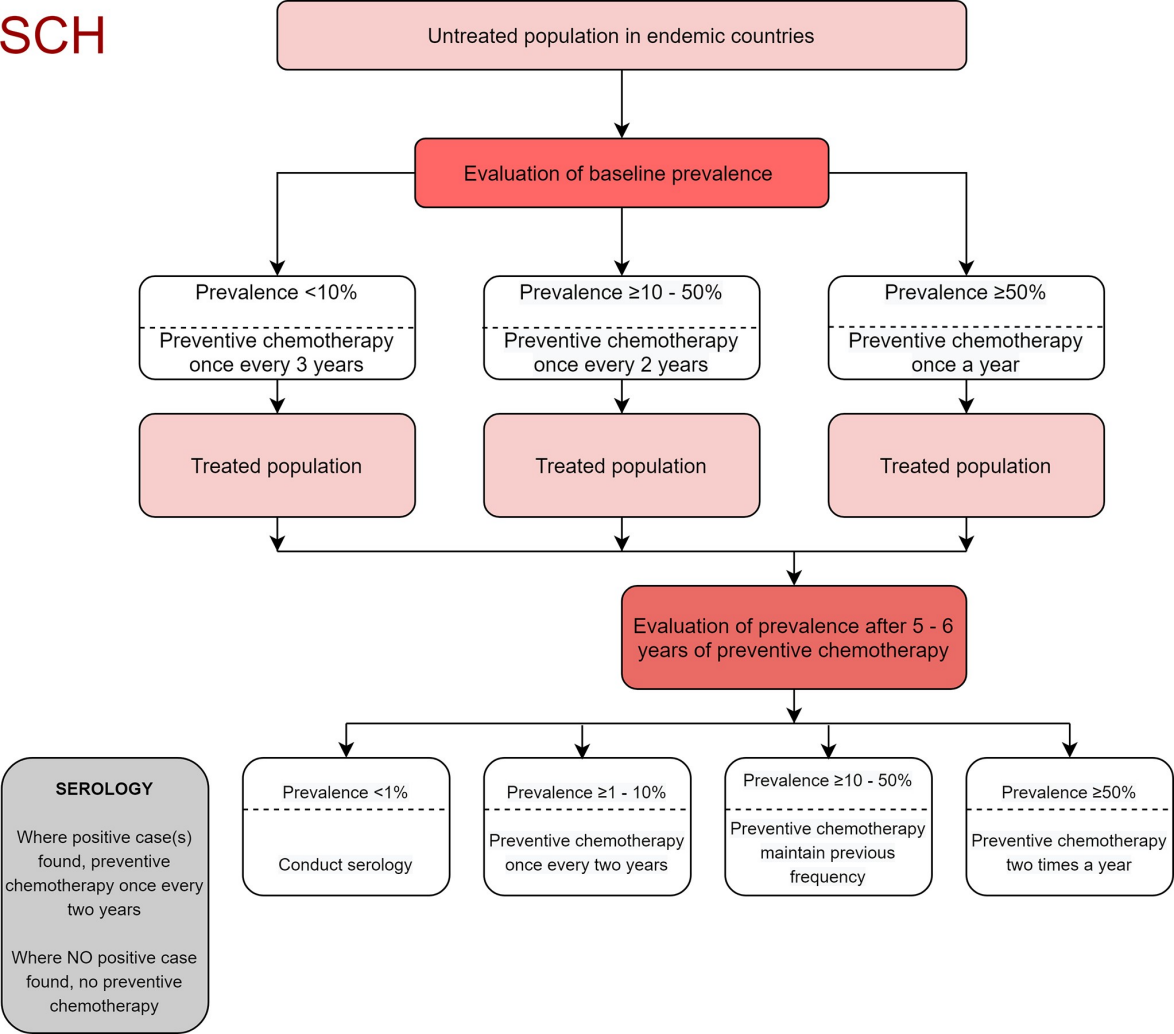
WHO guidelines decision tree for Schistosomiasis after five to six years of deworming.

Heterogeneity within counties for schistosomiasis indicated that prevalence was zero in ten counties, below 1% in four, between 1% and 10% in five and above 10% in only Busia County. Again noting that survey power being for the overall NSBD geographic area rather than county-level, plus the above mentioned recommendation for some new sites to be included in future surveys of schistosomiasis, means that care must be exercised before making a full recommendation that MDA treatment frequencies for schistosomiasis could change. However, again this picture indicates a lot of potential that such decisions may be able to be arrived at in future. According to the WHO guidelines for schistosomiasis, these decisions could in future be as follows: counties with zero prevalence could potentially consider stopping MDA, and counties with prevalence of below 1% would require serological surveys to determine cases of infection [5]; if from these serological surveys, positive cases are found then MDA would be recommended once after every two years. Counties with prevalence ranging between 1 to <10% for *S*. *mansoni* and *S*. *haematobium* respectively, could potentially conduct MDA once after every two years. Any county with prevalence of between 10 to < 50% would be warranted to maintain the previous MDA schedule of once every year (Fig 5, [5]). The other important observation to note from these low levels of schistosomiasis prevalence is that future large-scale surveys to assess schistosomiasis will not be very effective, as it will not be possible to power them to detect disease. For a highly focal infection it is instead likely that recent evidence of where schistosomiasis is in the country will be brought to bear, possibly with a change of surveying strategies towards more focal mapping (precision mapping) in the known endemic areas.

### WASH factors and their association with STH and schistosomiasis

Examination of the school and household WASH infrastructure as well as children’s behaviour and the infection prevalence was done across two domains; (i) helminth species, and (ii) WASH exposure levels i.e. individual-level, household-level and school-level exposures. Generally, coverage and access to key WASH indicators at home and school was relatively good, but was not always associated with lower infection prevalence and the associations were not always consistent between helminth species, likely reflecting both the different pathways of exposure between the species [32] and also some of the complexities in measuring WASH associations with helminth outcomes. Other studies have investigated the association between WASH and STH or schistosome species in Kenya [32–36], but few of them if any had assessed these associations for both individual, household and school level exposures at a national-level.

Although no significant associations between the WASH factors and any of the evaluated schistosomiasis prevalence were reported, driven primarily by lack of sufficient observations of schistosomiasis-positive individuals to measure these associations, past studies have investigated and reported possible significant risk factors associated with schistosomiasis [34,35,37–41].

The association of *A*. *lumbricoides* with soil-eating behaviour is not surprising since it is typically acquired through ingestion of helminth egg-contaminated food or untreated water, and so eating of soil (presumably contaminated soil) exposes individuals to high risk of *A*. *lumbricodes* as well as *T*. *trichiura* infections [42]. Walking bare foot has been previously reported to mostly increase the risk of hookworm infections since hookworm, unlike *A*. *lumbricoides*, is primarily acquired through skin contact especially walking bare footed on contaminated soil [43–45]. However, a recent study on a sub-cohort of school pupils participating in the Kenyan NSBD program similarly found that children not wearing shoes were equally predisposed to *A*. *lumbricoides* infection [32]. Additionally, we found that younger children (<5 years), were 4-fold more likely to be infected with STH while those in the SAC age group (5–14 years) were 3-fold more likely to be infected. Whilst traditionally children below five years were excluded from treatment due to them not attending school and a lack of suitable drug formulation, this finding reiterates the need to widen efforts within the deworming program to cover the PSAC cohort in Kenya. Even though helminth control efforts have been traditionally directed towards SAC, this finding echoes other evidence from within and outside Kenya over the past few years of the high risk of STH infections among PSAC, hence the need to include and strengthen deworming activities in this age group [46–48]. Importantly, a higher infection prevalence among this particular age group indicates the underlying risk of infection in a given area, which then become visible when a particular vulnerable age group is not treatment.

The study findings at household level of high number of household occupants, as well as children with no or low level of parents/guardians education being at significant risk of STH infections, points to possible increased risk from household overcrowding, which, especially in the rural areas could feasibly result in poor household hygiene practices and inadequate latrine facilities and safe water for drinking. When parents/guardians have no or low level of education, then the level of hygiene education they offer to their children might also be limited. Both these features are markers of general poverty and thus may be reflecting this well-established influencer of STH and schistosomiasis infection. From this result, it is important for control programs to emphasize continual strengthening of community-wide health education programs delivered through schools, health centres, places of worship, village/community meetings and such like channels to improve on the community understanding on STH control and prevention measures [49–53].

At school level, self-reported school absenteeism was the only notable significant risk factor, showing a gradient association with STH infections specifically *A*. *lumbricoides*, implying that, whilst one-day absence was not significantly associated with increased odds of STH infections, two-day and more than two-day absence were increasingly significantly associated with these infections. This result is consistent with previous studies that had found strong association between school absenteeism and *A*. *lumbricoides* and hookworm infections but not *T*. *trichiura* infection [54–56]. Whereas this study reports significant association between STH infections and school absenteeism, most studies have acknowledged difficulty in exclusively quantifying the effect of STH infections on school absenteeism, mainly attenuated by the low levels of infection intensity in the sample population [57–59]. However, a post-trial non-randomized study has previously shown that pupils with mostly moderate-to-heavy STH infections especially for *A*. *lumbricoides* were found to miss up to 2.4% of school days during follow-up periods compared to their uninfected counterparts [60]. Our study provides associational evidence of an effect of STH infections on school absenteeism among SAC. Evidence from robust randomized control trials may be needed to conclusively assess the effect of STH infections on key educational outcomes [59]. However, such intervention trials may be impossible to conduct over sufficient time periods to assess deworming impacts in the manner that such programs are delivered in real-world settings (i.e., repeated rounds administered throughout childhood) [61].

Additionally, the study found some factors to be protective against STH infections. Availability of tissue/newspaper/water for anal cleansing at home significantly lowered the odds of STH infections especially for *A*. *lumbricoides*. Proper use of tissue or water for anal cleansing has previously been reported as an important deterrent to STH infections [62]; if the cleansing material is used improperly by children or if inconsistently or completely unavailable, then anal cleansing after defecation may be done using one’s hand, which can lead to fecal contamination of the hands and thus exposure to helminth infection [32,63]. Children who reported always use of school drinking water were found to have lower odds of *T*. *trichiura*. Availability of drinking water to children especially when in school is key in controlling STH infections especially *A*. *lumbricoides* and *T*. *trichiura* since these infections are transmitted mainly through ingestion of contaminated food items like vegetables, fruits or drinking water [64]; this finding is consistent with other previous studies conducted among primary school children [8,32,65]. Children from households possessing assets such as television sets and electricity had lower odds of any STH infection. These household assets like television sets can be viewed as sources of information and can be used to increase the spread of STH advocacy, awareness and control messages through mass media adverts and talk shows [66]. More broadly than this, these are variables associated with increased household wealth relative to those households that do not have such items, providing more information on the likely underlying associations of these diseases with poverty. Interventions aimed at improving hygiene practices like proper anal cleansing after defecation, and access to safe drinking water, as well as continuing socioeconomic improvements, should be highlighted as recommendations for the long-term control of STH infections.

### Study limitations

This study was not without limitations. First, individual and household WASH indicators were self-reported by the sampled children at school and not directly collected or observed at home, thus this could potentially lead to reporting bias especially where children are young to recall some of the questions or provide the answer they believe the interviewer wants to hear. Secondly, since the infection prevalence has substantially reduced following several rounds of MDA delivered in the past years, building of robust multivariable models was limited by low prevalence (resulting in few positive values needed to run the models) of infection at Year 6 of the program. Thirdly, whilst the use of purposively sampling of schools within counties was deemed adequate since only schools with highest STH prevalence were targeted, representativeness and generalizability of our results might have been reduced by use of this sampling technique. Lastly, the program used the Kato-Katz technique for examination of STH and *S*. *mansoni* eggs in line with the WHO guidelines for examination of these infections in high endemic settings [16], however, this technique has been proven to be less sensitive in low endemic areas [26]. Therefore, our prevalence estimates could have under-estimated the true population prevalence.

## Conclusions

After five rounds of treatment, this impact assessment shows low levels of both STH and schistosomiasis in the National School-Based Deworming Program in Kenya. *Ascaris lumbricoides* is still the leading STH infection followed by *T*. *trichiura* and hookworm among SAC populations. The northern counties, Wajir and Garissa, showed zero prevalence of STH and schistosomiasis, an indication that there may be no ongoing biological transmission of the infections in the region. There was observed infection heterogeneity within counties, warranting careful investigation from this time going forward to determine whether reductions in treatment frequency can be proposed in some counties in future. Our assessment of the individual, household and school WASH practices and behaviour on STH and schistosomiasis prevalence suggested mixed associations and differed across individual helminth species; this is expected given the different mechanisms of infections. Based on the survey findings, the Kenyan NSBD program may wish to adopt county-level treatment frequencies based on the WHO guidelines, prioritize strategies to increase treatment coverage amongst the PSAC population, and incorporate integrated control approaches emphasizing health education and WASH interventions to the communities and schools.

## Supporting information

S1 Table World Health Organization (WHO) intensity thresholds for light, moderate and heavy infections with *Ascaris lumbricoides*, *Trichuris trichiura*, hookworm and schistosomes. (DOCX)
