# Correction: Prevalence, intensity and associated risk factors of soil-transmitted helminth and schistosome infections in Kenya: Impact assessment after five rounds of mass drug administration in Kenya

**DOI:** 10.1371/journal.pntd.0010550

**Published:** 2022-06-15

**Authors:** Collins Okoyo, Suzy J. Campbell, Katherine Williams, Elses Simiyu, Chrispin Owaga, Charles Mwandawiro

Table 2 contains some incorrect values regarding the number of schools and children examined in previous surveys. These errors affected the findings as reported in Table 6. Please see the correct Tables 2 and 6 below.

**Table 2:** Overall prevalence % (95%CI), mean intensity epg (95%CI) of infections and relative reductions (RR) % (p-value) among school children in Kenya after five rounds of MDA.

Survey	No. schools (children) surveyed	STH combined	Hookworm	*A*. *lumbricoides*	*T*. *trichiura*	*S*. *mansoni*	*S*. *haematobium*
**Prevalence, % (95%CI)**							
Year 1 Baseline*	173 (18,626)	32.3 (30.0–34.8)	15.4 (13.6–17.6)	18.1 (15.8–20.7)	6.7 (5.4–8.2)	2.4 (1.5–4.1)	18.0 (13.0–24.9)
Year 3 *	173 (18,199)	16.4 (14.4–18.6)	2.3 (1.8–3.0)	11.9 (10.2–13.9)	4.5 (3.4–6.0)	1.7 (0.8–3.6)	7.9 (3.8–16.2)
Year 5 *	172 (18,207)	13.5 (11.6–15.7)	1.3 (1.0–1.6)	9.6 (8.0–11.5)	4.1 (3.1–5.5)	2.0 (1.2–3.2)	3.9 (1.7–9.0)
Year 6 Evaluation^$^	100 (9,801)	12.9 (10.4–16.1)	1.0 (0.6–1.5)	9.7 (7.5–12.6)	3.6 (2.2–5.8)	2.2 (1.2–4.3)	0.3 (0.1–1.0)
RR (Y1Baseline–Y6Evaluation)	-	61.7 (p<0.001)	93.6 (p<0.001)	52.9 (p<0.001)	42.7 (p = 0.006)	7.9 (p = 0.779)	98.5 (p<0.001)
**Average Intensity, epg (95%CI)**							
Year 1 Baseline*	173 (18,626)	-	63 (50–81)	1659 (1378–1998)	33 (11–105)	14 (5–41)	20 (11–39)
Year 3 *	173 (18,199)	-	8 (5–14)	960 (801–1151)	17 (11–26)	6 (2–16)	7 (3–16)
Year 5 *	172 (18,207)	-	10 (5–19)	917 (750–1121)	16 (10–26)	5 (3–10)	4 (1–12)
Year 6 Evaluation^$^	100 (9,801)	-	6 (2–16)	741 (535–1027)	15 (8–27)	12 (5–31)	0 (0–1)
RR (Y1Baseline–Y6Evaluation)	-	-	90.7 (p<0.001)	61.1 (p<0.001)	58.3 (p = 0.201)	13.4 (p = 0821)	99.3 (p<0.001)

*Indicates surveys done under Year 1 (Y1), Year 3 (Y3) and Year 5 (Y5) monitoring and evaluation and included 200 schools in four regions [1–3]

^$^Indicates surveys done under this current assessment and included 100 schools in six regions

**Table 6:** Prevalence % (95%CI) and year 1 (Y1) to year 6 (Y6) relative reductions (RR) % (p-value) of light, moderate and heavy intensity of infections among school children in Kenya after five rounds of MDA.

Infections	Total children examined (Total positive)	Light infections^#^			Moderate infections^#^			Heavy infections^#^			Moderate-heavy infections^#^		
		n	Calculated using total children examined as denominator*	Calculated using total positives as denominator**	n	Calculated using total children examined as denominator*	Calculated using total positives as denominator**	n	Calculated using total children examined as denominator*	Calculated using total positives as denominator**	n	Calculated using total children examined as denominator*	Calculated using total positives as denominator**
**STH infections:**													
STH combined													
Y1 baseline	18,626 (6,274)	4,435	23.7 (22.0–25.5)	70.7 (67.3–74.2)	1,808	9.8 (8.3–11.4)	28.8 (25.6–32.5	31	0.2 (0.1–0.5)	0.5 (0.2–1.5)	1,839	9.9 (8.5–11.6)	29.3 (26.1–33.0)
Y6 evaluation	9,801 (1,274)	843	8.6 (6.9–10.8)	59.1 (51.3–68.0)	392	4.0 (2.9–5.4)	27.5 (22.0–34.3)	7	2.0 (0.9–4.2)	0.5 (0.2–1.1)	399	6.0 (4.4–8.2)	28.0 (22.4–34.9)
RR% (p-value)	-		63.9% (p<0.001)	16.4% (p = 0.014)		58.8% (p<0.001)	4.7% (p = 0.651)		*Increased* (11.8%, p<0.001)	0.7% (p = 0.992)		39.6% (p<0.001)	4.6% (p = 0.653)
*A*. *lumbricoides*													
Y1 baseline	18,626 (3,843)	2,086	11.2 (10.0–12.7)	54.3 (51.1–57.7)	1,757	9.5 (8.0–11.2)	45.7 (42.5–49.2)	**-**	-^$^	-^$^	1,757	9.5 (8.0–11.2)	45.7 (42.5–49.2)
Y6 evaluation	9,801 (935)	561	5.8 (4.5–7.5)	60.0 (54.4–66.2)	371	3.9 (2.8–5.3)	39.7 (34.3–45.9)	3	1.9 (0.9–4.2)	0.3 (0.1–1.0)	374	5.7 (4.1–7.9)	40.0 (34.5–46.3)
RR% (p-value)	-		47.9% (p<0.001)	*Increased* (10.5%, p = 0.062)		59.1% (p<0.001)	13.2% (p = 0.065)		-^$^	-^$^		39.2% (p = 0.002)	12.5% (p = 0.082)
Hookworm													
Y1 baseline	18,626 (2,856)	2,809	14.9 (13.0–17.1)	98.4 (95.6–99.7)	33	0.2 (0.1–0.3)	1.2 (0.7–1.8)	14	0.1 (0–0.1)	0.5 (0.3–0.9)	47	0.3 (0.2–0.4)	1.6 (1.1–2.4)
Y6 evaluation	9,801 (94)	91	2.8 (1.6–4.8)	96.8 (92.3–98.2)	0	0	0	3	0 (0–0.1)	3.2 (0.7–13.6)	3	0 (0–0.1)	3.2 (0.7–13.6)
RR% (p-value)	-		81.3% (p<0.001)	1.6% (p = 0.412)		100% (p<0.001)	100% (p<0.001)		58.5% (p = 0.279)	*Increased* (551.1% (p = 0.019)		87.6% (p = 0.007)	*Increased* (93.4% (p = 0.387)
*T*. *trichiura*													
Y1 baseline	18,626 (1,169)	1,112	6.0 (4.8–7.6)	95.1 (92.0–98.4	40	0.2 (0.1–0.3)	3.4 (2.4–4.8)	17	0.1 (0–0.7)	1.5 (0.2–10.1)	57	0.3 (0.2–0.6)	4.9 (2.5–9.4)
Y6 evaluation	9,801 (346)	322	5.2 (3.5–7.8)	93.1 (90.4–95.8)	23	0.2 (0.1–0.5)	6.6 (4.4–10.1)	**1**	0 (0–0.1)	0.3 (0–2.2)	24	0.2 (0.1–0.5)	6.9 (4.7–10.3)
RR% (p-value)	-		13.4% (p = 0.458)	2.2% (p = 0.337)		*Increased* (11.4%, p = 0.770)	*Increased* (94.3%, p = 0.012)		88.6% (p = 0.125)	80.1% (p = 0.253)		18.4% (p = 0.674)	*Increased* (42.3%, p = 0.367)
**Schistosome infections:**													
Any schistosome													
Y1 baseline	18,626 (701)	351	1.9 (1.7–2.1)	50.1 (38.1–65.8)	130	0.7 (0.4–1.4)	18.5 (15.7–21.6)	219	1.2 (0.6–2.2)	31.2 (21.6–45.2)	132	0.7 (0.6–0.8)	18.8 (16.0–21.9)
Y6 evaluation	9,801 (223)	91	0.9 (0.7–1.1)	40.8 (31.2–53.3)	74	0.8 (0.4–1.6)	34.6 (29.6–40.4)	58	0.6 (0.4–0.8)	26.0 (17.6–38.5)	26	0.3 (0.1–0.4)	11.7 (7.8–16.6)
RR% (p-value)	-		52.6% (p<0.001)	18.5% (p = 0.175)		*Increased* (8.2%, p = 0.819)	*Increased* (87.0%, p = 0.276)		50.0% (p = 0.040)	16.7% (p = 0.412)		57.1% (p = 0.007)	37.8% (p = 0.847)
*S*. *mansoni*													
Y1 baseline	18,626 (450)	183	1.0 (0.7–1.5)	40.7 (27.2–60.9)	130	0.7 (0.4–1.4)	28.9 (21.1–39.6)	137	0.7 (0.3–1.8)	30.4 (17.3–53.5)	267	1.4 (0.7–2.9)	59.3 (45.0–78.2)
Y6 evaluation	9,801 (214)	85	0.9 (0.5–1.5)	39.7 (30.2–52.2)	74	0.8 (0.4–1.6)	34.6 (29.6–40.4)	55	0.6 (0.4–0.7)	25.7 (16.9–39.0)	129	1.3 (1.1–1.6)	60.3 (50.4–72.1)
RR% (p-value)	-		10.0% (p = 0.716)	2.3% (p = 0.904)		*Increased* (10.3%, p = 0.776)	*Increased* (19.7%, p = 0.276)		14.3% (p = 0.063)	15.6% (p = 0.558)		7.1% (p = 0.378)	*Increased* (1.6%, p = 0.905)
*S*. *haematobium*													
Y1 baseline	1,399 (252)	169	12.1 (10.4–13.9)	67.1 (56.8–79.2)	**-**	-^$^	-^$^	83	5.9 (3.5–10.1)	32.9 (23.5–46.3)	83	5.9 (3.5–10.1)	32.9 (23.5–46.3)
Y6 evaluation	3,417 (9)	6	0.2 (0.1–0.4)	66.7 (49.0–90.7)	**-**	-^$^	-^$^	3	0.1 (0–0.4)	33.3 (18.0–61.7)	3	0.1 (0–0.4)	33.3 (18.0–61.7)
RR% (p-value)	-		98.3% (p<0.001)	0.6% (p = 0.974)	**-**	-^$^	-^$^		98.5% (p<0.001)	*Increased* (1.2%, p = 0.973)		98.5% (p<0.001)	*Increased* (1.2%, p = 0.973)

-^$^ Indicate that prevalence of intensity was not assessed at that particular cut-off point

n; indicates the number positive for each intensity class

^**#**^Prevalence of each intensity class was calculated using two approaches: 1)* when taking the denominator as the overall number of children examined, and 2)** when taking the denominator as the total number of positive-children for each infection. The use of these two approaches enabled us to conveniently compare the morbidity due to these infections and for easy comparison to other studies.
