# Supplementary material for: Prevalence, intensity and associated risk factors of soil-transmitted helminth and schistosome infections in Kenya: Impact assessment after five rounds of mass drug administration in Kenya
Source: PLoS Negl Trop Dis. 2020 Oct 7;14(10):e0008604. doi: 10.1371/journal.pntd.0008604 (PMC7540847; doi:10.1371/journal.pntd.0008604)
Supplement: S1 Table — (DOCX) [file pntd.0008604.s001.docx]

**Supplementary File:**

**Table S1: World Health Organization (WHO) intensity thresholds for light, moderate and heavy infections with *Ascaris lumbricoides*, *Trichuris trichiura*, hookworm and schistosomes**

| **Helminth*** | **Intensity Threshold** | | |
| --- | --- | --- | --- |
|  | **Light** | **Moderate** | **Heavy** |
| *A. lumbricoides* | 1 – 4999 epg | 5000 – 49999 epg | ≥ 50000 epg |
| *T. trichiura* | 1 – 999 epg | 1000 – 9999 epg | ≥ 10000 epg |
| Hookworms | 1 – 1999 epg | 2000 – 3999 epg | ≥ 4000 epg |
| *S. mansoni* | 1 – 99 epg | 100 – 399 epg | ≥ 400 epg |
| *S. haematobium* | 1 – 50 eggs/10ml urine | - | ≥ 50 eggs/10ml urine |

*These intensity thresholds were adopted from a WHO report of 2002 on helminth control in school age children [**20**]
